# Integrative SAXS and AFM analysis of engineered carbohydrate‐active enzyme assemblies with tunable spatial organization

**DOI:** 10.1002/pro.70649

**Published:** 2026-06-15

**Authors:** Iker Pardo Larrabeiti, Manuel Eibinger, Jeremy Esque, Stéphanie Pradeau, Sébastien Fort, Sarah Moraïs, Itzhak Mizrahi, Edward A. Bayer, Bernd Nidetzky, Cédric Y. Montanier, Pierre Roblin, Claire Dumon

**Affiliations:** ^1^ TBI, Université de Toulouse, CNRS, INRAE, INSA Toulouse France; ^2^ Institute of Biotechnology and Biochemical Engineering Graz University of Technology Graz Austria; ^3^ University of Grenoble Alpes, CNRS, CERMAV Grenoble France; ^4^ Department of Life Sciences Ben‐Gurion University of the Negev Be'er Sheva Israel; ^5^ Department of Biomolecular Sciences The Weizmann Institute of Science Rehovot Israel; ^6^ University of Toulouse, CNRS, LGC Toulouse France

**Keywords:** AFM, cellulase, endoglucanase, glycoside hydrolase, SAXS, spatial proximity, xylanase

## Abstract

Cellulosomes are multi‐enzyme assemblies whose catalytic efficiency depends on the spatial organization of their components. However, their pronounced conformational flexibility has precluded quantitative characterization of inter‐enzyme distances and overall topology. Here, we present a methodological framework to tailor and analyze the architecture of constrained multi‐enzyme complexes composed of two endoglucanases *At*Cel8A and *At*Cel9R and one xylanase *At*Xyn11A by fixing enzyme positions using the Jo‐In scaffold. This approach enables generation of defined assemblies structurally characterized by small‐angle X‐ray scattering (SAXS) and/or atomic force microscopy (AFM). SAXS analysis of the two‐glucanase complexes reveals distinct scattering profiles corresponding to different degrees of compaction depending on the enzyme spatial arrangement. Complementary AFM imaging of bifunctional assemblies supports SAXS derived models at the single‐particle level, reinforcing the robustness of the proposed workflow. The three‐enzyme assemblies' SAXS measurements distinguish different constructions while showing relatively homogeneous radii of gyration (53 ± 2 Å) and maximum dimensions of 180 to 200 Å. Atomistic modeling using two independent approaches, DADIMODO and BILBO‐MD, converges toward consistent average spatial organizations with constrained interdomain distance ranges, and consistent quantitative parameters validating the structural models. Altogether, the results establish a quantitative framework for designing tailored multi‐enzyme assemblies. Jo‐In scaffold provides a versatile tool for structural analysis of modular, dynamic protein complexes, advancing our understanding of structure–function relationship in multi‐enzyme systems.

## 1 INTRODUCTION

Multi‐domain proteins constitute over half of all proteomes and up to 70% of eukaryote proteins, highlighting modularity's importance in protein evolution (Han et al., 2007). This principle is exemplified by glycoside hydrolases (GHs), which frequently combine catalytic domains with non‐catalytic carbohydrate‐binding modules (CBMs) (Drula et al., 2022). Increasing evidence indicates that flexible linkers connecting these domains modulate enzyme activity, substrate accessibility, and interdomain synergies (Forsberg et al., 2023). Nature exploits this modularity at higher organizational levels, as seen in the cellulosome, a specialized multi‐enzyme complex that efficiently degrades insoluble plant cell wall polysaccharides. Here, dockerin modules bind specifically to cohesin‐bearing scaffoldins, colocalizing enzymes in close proximity to both the substrate and one another (Artzi et al., 2017). The scaffolding harbors a CBM3a that targets crystalline cellulose while adjacent linkers influence its positioning (Yaniv et al., 2013). The cellulosome has inspired the concept of “designer cellulosomes” (DCs) where orthogonal cohesin–dockerin interactions enable construction of tailored multi‐enzyme assemblies (Fierobe et al., 2001) which typically outperform the equivalent set of free enzymes (Vodovnik & Lindič, 2025).

Several studies have probed how enzyme spatial organization within DCs affects catalytic performance. Key variables include the scaffolding linker length (Caspi et al., 2009; Molinier et al., 2011; Vazana et al., 2013); enzyme positioning, which can determine synergy or anti‐synergy (Moraïs et al., 2010); CBM3a placement for substrate targeting (Stern et al., 2015); scaffolding geometry (Mingardon et al., 2007) and the interplay between free and scaffold‐bound enzymes (David et al., 2025). Many of these studies relied on major modifications in scaffolding architecture to tune inter‐enzyme distances and relative orientations. Collectively, suggesting that improved performance arises from the close spatial proximity of enzymes and from scaffolding flexibility. Indeed, catalytic domains would adapt to the heterogeneous surface of insoluble substrates while limiting product diffusion and thereby triggering synergistic effects.

Structural and biophysical approaches on both cellulosomes and DCs have been extensively used to better correlate efficiency with the enzyme's spatial organization. Small‐angle X‐ray scattering (SAXS) enables solution‐state analysis of complexes, providing information on conformational fluctuations, overall dimensions, and compactness. SAXS studies from single enzymes (Hammel et al., 2004) to di‐ (Currie et al., 2012; Czjzek et al., 2012; Hammel et al., 2005; Molinier et al., 2011) and tri‐valent DCs (Dorival et al., 2022) have revealed coexisting compact and extended conformations. Complementary techniques including molecular modeling (Bomble et al., 2011), cryogenic electron microscopy (cryo‐EM) (García‐Alvarez et al., 2011) and atomic force microscopy (AFM) (Eibinger et al., 2020) have confirmed this conformational heterogeneity, which is primarily driven by intrinsic disorder in the linker regions.

Therefore, it is hypothesized that the cellulosome flexibility plays a key role in catalytic efficiency by enabling dynamic enzyme positioning. This raises the question of whether enzymes form transient associations or maintain variable inter‐enzyme distances during hydrolysis. A promising approach to address this question involves fixing enzymes in well‐defined, rigid spatial configurations to systematically investigate the effect of inter‐enzyme distance on catalysis. We achieved this using two engineered protein fragments, Jo and In, that spontaneously form an irreversible isopeptide bond creating a complex Jo‐In also called the biomolecular welding tool (Bonnet et al., 2017). It enables rigid positioning of enzymes by genetically fusing each enzyme at either one extremity of the Jo or In component. Upon mixing, Jo and In spontaneously and covalently bond, locking the relative orientation of the fused enzymes while maintaining their independent folding and catalytic activity. This strategy has been successfully applied to connect pairs of GHs (Enjalbert et al., 2020) or a GH with a CBM (Badruna et al., 2021) at fixed spatial arrangements without impairing their function.

In this study, we probe the structural organization between three GHs assembled into six distinct complexes via Jo‐In coupling using AFM and SAXS‐constrained modeling. Inspired by a “dissect‐and‐reconstruct” strategy (Smith et al., 2017) where enzymatic components are characterized prior to reconstruction of the multi‐enzyme system, we characterized one‐, two‐ and three‐enzyme assemblies that mimic DCs to gain insight into the three‐dimensional structural organization. Two endo‐1,4‐β‐glucanases (*At*Cel9R and *At*Cel8A) and one endo‐1,4‐β‐xylanase (*At*Xyn11A), from the cellulosomal bacteria *Acetivibrio thermocellus* (formerly termed *Clostridium thermocellum*), were used and their activity once in complexes was validated. A workflow integrating both Minimal Ensemble Search (MES) from BILBO‐MD and DADIMODO software to assess flexibility was implemented to characterize the three enzyme complexes. AFM experiments complemented and corroborated the modeling multi‐domain refinement when the two glucanases were in complex. Overall, this approach enabled a systematic estimation of average inter‐enzyme distances, providing structural insight into these multi‐enzyme complexes.

## 2 RESULTS

### 2.1 Multi‐enzymatic assembly strategy

Among the identified *A. thermocellus* cellulosomal enzymes (Raman et al., 2011; Yoav et al., 2017), we selected *At*Cel9R, *At*Cel8A (Dorival et al., 2022; Stern et al., 2015) and *At*Xyn11A (Moraïs et al., 2010) that are produced by the bacteria during plant cell wall degradation. Both Cel8A and Cel9R cellulases have been overexpressed in *E. coli*, and well characterized with crystal structures at 0.94 Å PDB: 1KWF (Guérin et al., 2002) and 2.00 Å PDB: 7UNP (Kuch et al., 2023), respectively. *At*Xyn11A displays a modular architecture consisting of a GH11 xylanase and a CBM from CBM6 family linked by a 20 amino acid linker (Fernandes & Ferreira, 1999; Hayashi et al., 1999). Although no 3D structure has been reported for this particular xylanase domain, members of the GH11 family are well described as a β‐jelly roll fold (Vardakou et al., 2008). The structure of *At*CBM6, however, has been published (Czjzek et al., 2001). These three cellulosomal enzymes operate in the same conditions (pH and temperature) and were already produced in designer cellulosomes constituting good candidates to explore multi‐enzyme assembly.

**FIGURE 1:**
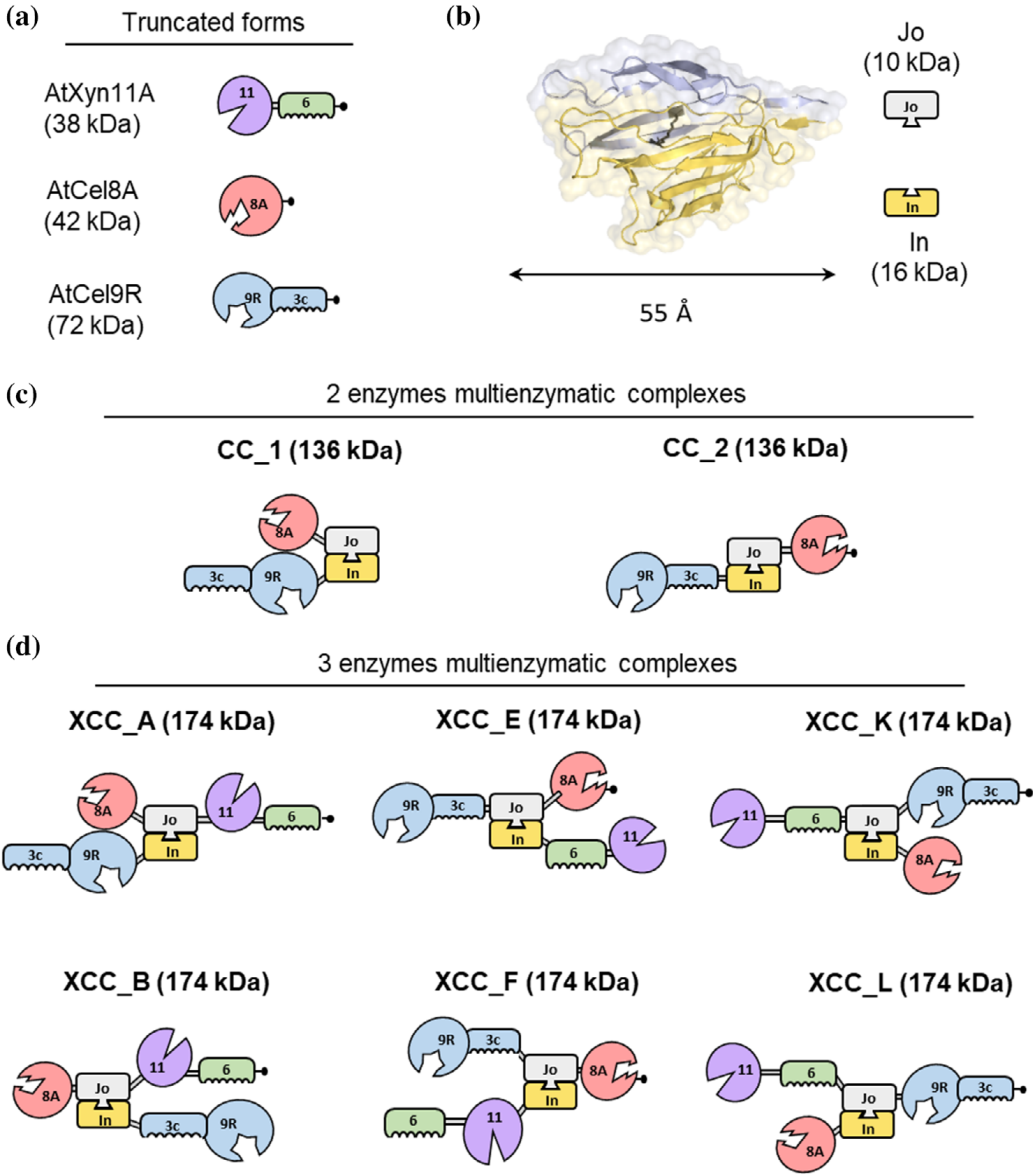
Schematic representation of the recombinant multienzymatic complexes analyzed in this study. (a) Truncated forms (lacking the dockerin and intermodular linker) of the glycoside hydrolases (GHs) and their carbohydrate‐binding module (CBM) used in the assemblies. Numbers indicate the GH family of the catalytic modules (GH11, GH8, GH9) or the CBM family (CBM6, CBM3c). (b) The Jo‐In biomolecular welding system composed of Jo (10 kDa) and In (16 kDa) domains. (c) Bifunctional multienzymatic complexes (CC_1 and CC_2; 136 kDa each) consisting of *At*Cel9R fused to the N terminus of In, combined with *At*Cel8A fused at either the N or C terminus of Jo. (d) Trifunctional multienzymatic complexes (6 XCC constructs labeled from A to L; 174 kDa each) generated using a chimeric Jo domain bearing two enzymes at its termini as shown in the Figure (with a C‐terminal His_6_ tag, designated –•) and an In domain fused to a third enzyme at either its N‐ or C‐terminus. Since the termini of In are structurally antiparallel to those of Jo, the enzymes appear on the right side of the In protein in the schematic, even though they are actually fused to its N‐terminus.

Unlike cellulosomal enzymes which naturally contain a dockerin module and a linker connected to the catalytic domain, Jo‐In‐mediated complexes comprise only catalytic modules and CBMs, to restrict the degree of conformational or dynamic freedom conferred by the linker (Dorival et al., 2022) and enabling precise enzyme positioning as shown in Figure 1a. Enzyme complexes were generated using the Jo‐In biomolecular welding tool, which allows the spontaneous formation of an intramolecular isopeptide bond between the Jo (K191) and In (N695) partner, yielding a rigid core (Figure 1b). By mixing Jo and In chimeric enzyme variants, we assembled multi‐enzyme complexes, in which each catalytic unit is covalently anchored at defined positions. In total, eight different complexes were produced, two of which contain Cel8A and Cel9R (named CC_1 and CC_2, shown in Figure 1c) and 6 complexes harboring the 3 enzymes together (from XCC_A to L) shown in Figure 1d.

### 2.2 Validation of enzyme activity in complexes

The catalytic activity of individual enzymes within the assemblies was assessed relative to their free forms. To circumvent potential limitations in active site accessibility arising from restricted conformational flexibility and inter‐enzyme proximity within the assemblies, small chromogenic substrates were employed. Xylanase and endoglucanases were assayed with 4‐nitrophenyl‐xylotrioside (X_3_‐*p*NP) and 4,6‐O‐(3‐Ketobutylidene)‐4‐nitrophenyl‐β‐D‐cellopentaoside (BPNPG5), respectively. Individual endoglucanase contributions could not be resolved within the complexes; therefore, only their combined activity was measured.

Activity measured on free enzymes confirmed that xylanase and endoglucanase exhibited strict substrate specificity on X3‐*p*NP and BPNPG5 respectively (Table 1). Enzyme assemblies showed similar activity on X3‐*p*NP ranging from 306 ± 5 to 383 ± 16 U/μM with XCC‐E exhibiting a 10% decrease in specific activity compared to the free xylanase. Similarly, the specific activities of *At*Cel8A and *At*Cel9R were 3.7 ± 0.2 U/μM and 0.5 ± 0.1 U/μM, respectively (Table 1). When *At*Cel8A, *At*Cel9R, and *At*Xyn11A were combined in solution, specific activity corresponded to the sum of the specific activities of the individual endoglucanases, indicating no synergistic interactions. Within assemblies, enzyme activities were comparable to those of the free enzymes, with the notable exception of XCC‐F, which exhibited a ≈20% increase in endoglucanase activity. Overall, the data demonstrate that the intrinsic enzymatic activity has been retained within the complexes.

**TABLE 1:** Specific activity of multi‐functional assemblies and free counterparts on X_3_‐*p*NP and BPNPG5.

U/μM	Free enzymes					Assemblies					
	Cel8A	Cel9R	Xyn11A	Cel8A‐Cel9R	Cel8A‐Cel9R‐Xyn11A	XCC‐A	XCC‐B	XCC‐E	XCC‐F	XCC‐K	XCC‐L
X3‐pNP	0^a^	0^a^	363 ± 16	1 ± 0	337 ± 14	366 ± 1	370 ± 1	306 ± 5	327 ± 9	383 ± 16	325 ± 13
BPNPG5	3.7 ± 0.2	0.5 ± 0.1	0 ± 0	4.2 ± 0.1	4.6 ± 0.4	4.3 ± 0.1	4.4 ± 0.1	4.8 ± 0.2	5.3 ± 0.1	4.4 ± 0.1	3.8 ± 0.3

^a^ *n* = 1.

### 2.3 SAXS analyses of individual enzymes

**FIGURE 2:**
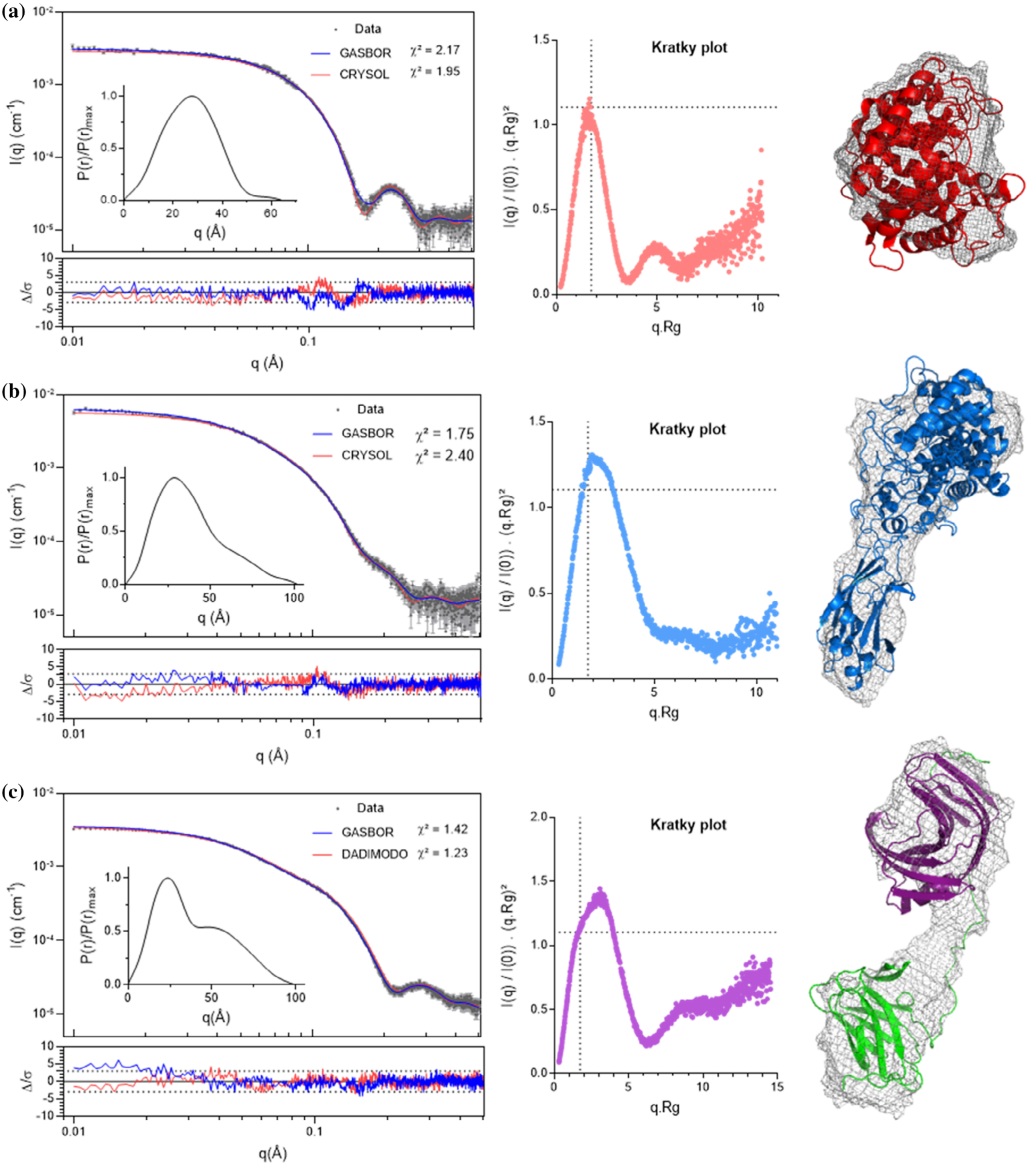
Experimental SAXS data are shown in gray dots from top to bottom for *At*Cel8A (a). *At*Cel9R (b). and *At*Xyn11A (c). In the inset, normalized autocorrelation (*P*(*r*)/*P*(*R*)_max_) function is displayed. Normalized Kratky are displayed in red for *At*Cel8A; blue for *At*Cel9R and violet/green for *At*Xyn11A. Two models are fitted to the raw data and normalized residuals for both fits are shown below each curve. An ab initio envelope reconstruction was generated using GASBOR (green curve) and a theoretical scattering curve from atomic models using CRYSOL or DADIMODO; the most representative model of *At*Xyn11A is displayed. *D*_max_, *R*_*g*_ and apparent molecular weight are displayed in Table 2 along with χ^2^ values for each model. Ab initio and all‐atom models were aligned using CIFSUP and displayed with color according to Kratky plot.

The free enzymes, *At*Cel8A, *At*Cel9R, and *At*Xyn11A, were analyzed by SAXS to apply the dissect‐and‐reconstruct strategy (Smith et al., 2017). SAXS analyses are summarized in Figure 2 while key parameters, including the radius of gyration (*R*_*g*_), maximum particle dimension (*D*_max_), and apparent molecular weight, are listed in Table 2. *At*Cel8A displays a compact and globular conformation in solution. The normalized Kratky plot exhibits the bell‐shaped profile with a main peak at *q*·*R*_*g*_ ≈1.73 and a secondary shoulder at higher q, indicating preferred intramolecular distances within the folded core (Figure 2a). The interpretation is consistent with the bell‐shaped *P*(*r*) function and supports a well‐folded architecture. The experimental curve fits closely with the crystallographic structure, as confirmed by CRYSOL fitting (*χ* ^2^ = 1.94), indicating good agreement across the measured q‐range. *At*Cel9R shows a broader *P*(*r*) distribution with *D*_max_ = 102 Å, and a Kratky profile revealing a globular core accompanied by asymmetric extensions (Figure 2b). The autocorrelation function supports the presence of a compact domain followed by an extended segment. This feature is consistent with the presence of a CBM3c, which remains covalently attached to the catalytic core through a linker. The SAXS data agree well with the available crystallographic structure. The scattering profile of *At*Xyn11A exhibits a two‐regime profile, characteristic of a multimodular protein (Figure 2c). The Kratky plot deviates from the ideal bell shape, showing a plateau at high *q*, indicative of partial flexibility between domains. The *P*(*r*) function reveals two distinct peaks, confirming a two‐domain organization, also revealing that the CBM6 adopts a spatially restricted conformation rather than a fully flexible one. Ab initio envelopes generated with GASBOR (NSD = 1.01) (Figure S1a) are consistent with the DADIMODO‐refined models (χ^2^ = 1.23–2.6), supporting a stable bimodular arrangement (Figure S1b).

**TABLE 2:** SAXS parameters derived from experimental scattering curves and modeling.

System	*R*_*g*_ (Å)	*D*_max_ (Å)	*M*_*w*_ (kDa)	CRYSOL	GASBOR	DADIMODO	MES (2 conformers)	
				X^2^	X^2^	X^2^ ^a^	X^2^ ^b^	%
Free enzymes								
AtCel8A	20.4 ± 0.2	64	38	1.95	2.17	–	–	–
AtCel9R	30.8 ± 0.2	100	66	2.4	1.75	–	–	–
AtXyn11A	29.0 ± 0.1	100	42	–	1.42	1.23	–	–
2 enzyme complexes								
CC_1	46.6 ± 0.5	172	124	–	–	1.60	–	–
CC_2	43.0 ± 0.2	130	142	–	–	1.17	–	–
3 enzyme complexes								
XCC_A	52.8 ± 0.2	187	195	–	–	1.35	2.82	58/42
XCC_B	53.6 ± 0.2	204	194	–	–	1.91	2.6	65/35
XCC_E	54.7 ± 0.2	190	194	–	–	1.22	1.38	75/25
XCC_F	53.7 ± 0.4	183	202	–	–	1.08	1.32	77/23
XCC_K	55.0 ± 0.5	180	191	–	–	1.23	3.05	30/70
XCC_L	53.7 ± 0.5	193	202	–	–	1.16	2.41	75/25

^a^ Chi^2^ values calculated with CRYSOL.

^b^ Chi^2^ values calculated with MULTI‐FOX.

### 2.4 SAXS characterization of bifunctional complexes

**FIGURE 3:**
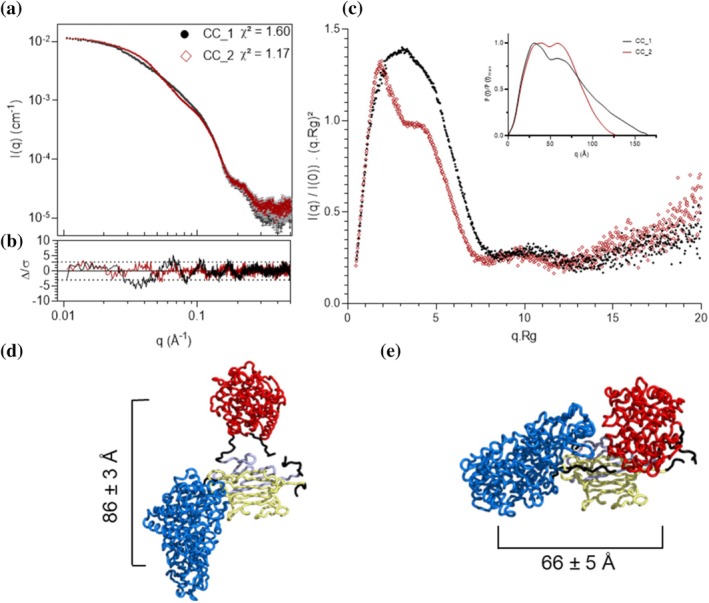
SAXS analysis and modeling of bifunctional complexes. (a) Experimental SAXS curves for CC_1 (black circles) and CC_2 (red diamonds), scaled to emphasize structural differences. χ^2^ values for the most representative DADIMODO‐generated models are indicated, with residuals shown in (b). (c) Dimensionless Kratky plots with autocorrelation functions inset. (d, e) Best‐fit models among five independent DADIMODO runs for CC_1 and CC_2, respectively, with mean distances between catalytic residues indicated. Cel9R is shown in blue, Cel8A in red, and Jo–In in gray/yellow according to Figure 1.

Intermediate assemblies containing the two endoglucanases (herein referred to as CC_1 and CC_2, in Figure 1) were next analyzed by SAXS. The corresponding scattering curves are shown in Figure 3a. Guinier analysis yielded *R*_*g*_ values of 46.3 Å for CC_1 and 43.2 Å for CC_2 (Table 2). Noticeable differences appeared in the intermediate q‐range (0.02 < *q* < 0.15 Å^−1^), suggesting distinct overall topologies between the two assemblies (Figure 3b). The Kratky plots emphasized these differences: CC_1 exhibited a more open, elongated conformation, while CC_2 appeared more compact (Figure 3c). Both assemblies retained features of well‐folded proteins but clearly deviated from ideal globularity, consistent with modular architectures. The corresponding *P*(*r*) functions provided additional structural insight. Although both complexes showed a main maximum around 58 Å, CC_2 exhibits a markedly smaller *D*_max_ than CC_1 (130 Å vs. 172 Å, respectively), in agreement with its lower *R*_*g*_ value (43.2 Å vs. 46.3 Å) and with the more compact profile observed in the normalized Kratky plot. Together, these parameters indicate that CC_2 adopts a more compact average organization, whereas CC_1 samples a more extended arrangement. Collectively, CC_1 and CC_2 exhibited *R*_*g*_ and *D*_max_ values consistent with previously reported bifunctional designer cellulosomes (Molinier et al., 2011).

All‐atom models were generated using DADIMODO to interpret the SAXS data at the atomistic level. For each complex, five independent optimization runs yielded *χ* ^2^ values of 1.61–2.80 for CC_1 and 1.17–1.32 for CC_2, indicating good agreement with experimental data (Figure S2). Particular emphasis was placed on the intermediate scattering region (*q* ≈ 0.05–0.15 Å^−1^), which is most sensitive to overall molecular shape and interdomain organization, thereby capturing the major structural differences between complexes. The most representative fits among the five generated are shown in Figure 3c, and the corresponding model ensembles in Figure S2. The catalytic core of the two endoglucanases adopted reproducible relative orientations across all runs, supporting the robustness of the resulting models. In contrast, the orientation of the CBM3c domain of *At*Cel9R remained inconsistent in both assemblies, with no convergence toward a unique spatial arrangement. The average interdomain distance between the two catalytic domains was 86 ± 3 Å for CC_1 and 66 ± 5 Å for CC_2, consistent with the differences inferred from *P*(*r*) and Kratky analyses. Ab initio reconstructions performed with GASBOR did not converge to a single consensus envelope (NSD >1), indicating conformational heterogeneity likely arising from the CBM3c mobility (Figure S3). As anticipated, these observations corroborated the divergent topologies in solution. These observations support the presence of multiple conformations in solution rather than a single defined structure. This structural variability prevented generation of a unique averaged shape but indicates that the Jo‐In linkage maintains local flexibility around the CBM region while constraining the global enzyme organization.

Overall, SAXS analyses demonstrate that both CC_1 and CC_2 form compact, well‐folded assemblies with distinct average spatial organizations: CC_2 corresponds to a more compact form, while CC_1 adopts a more extended configuration. These observations establish that modest variations in Jo–In fusion geometry are sufficient to generate measurable differences in molecular organization, thereby highlighting the sensitivity of SAXS to probe structural constraints within multi‐enzyme assemblies.

### 2.5 AFM characterization of bifunctional enzymatic complexes

**FIGURE 4:**
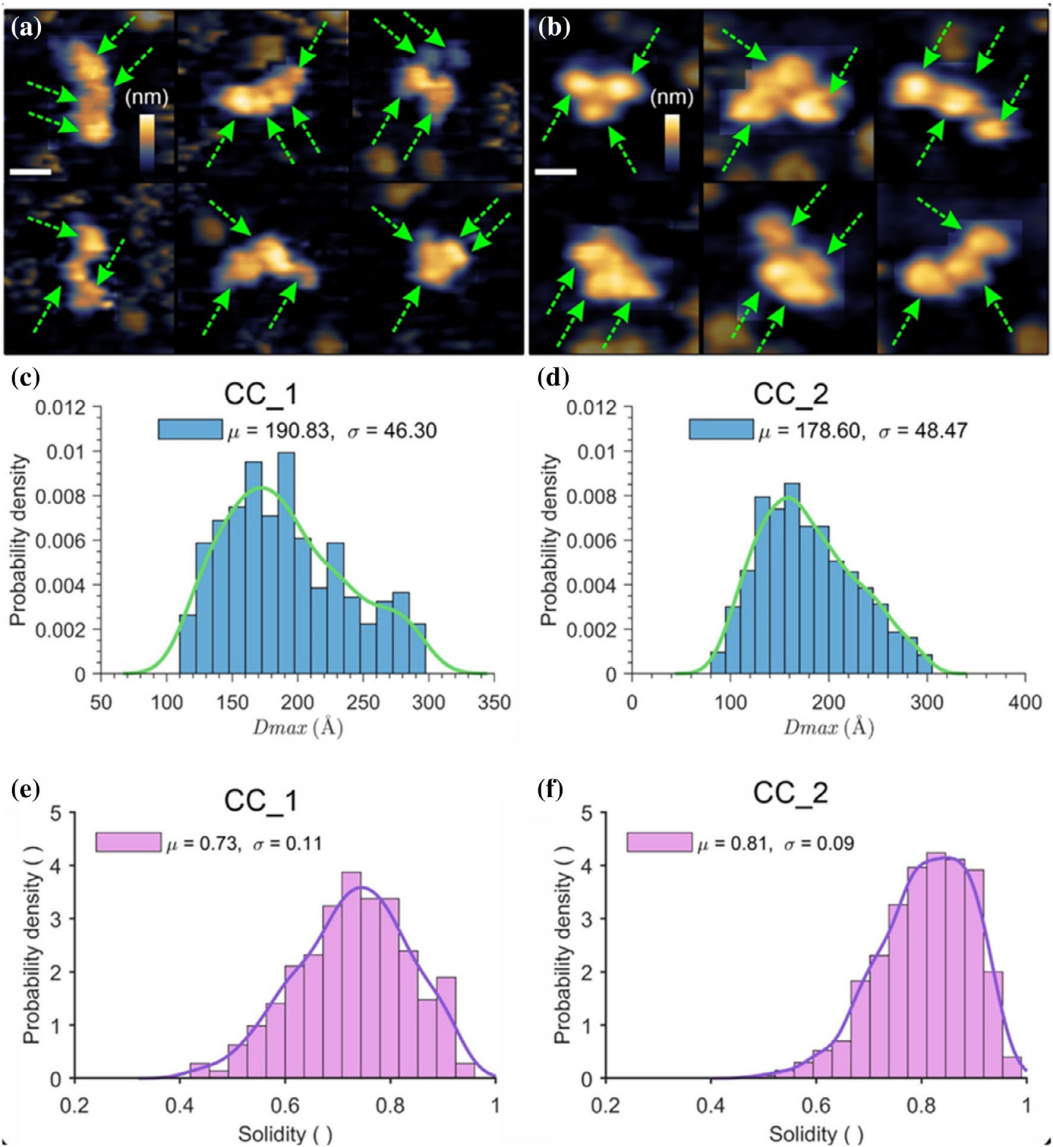
AFM imaging. Representative particles of CC_1 (a) and CC_2 (b) adsorbed on HOPG. CC_1 commonly displays elongated, anisotropic shapes, whereas CC_2 predominantly appears compact and near‐circular. The right panels show an alternative conformation, included to illustrate the natural variability in particle shape for each construct. Individual subunits, when visible, are indicated by green arrows; no subunit assignment was attempted. For easier visualization, the background intensity in panels a and b has been reduced. (c, d) Distribution of *D*_max_ for CC_1 359 particles (c) and 1108 CC_2 particles (d) on HOPG. (e, f) Distribution of solidity values for CC_1 (e) and CC_2 (f). Colored lines above the histograms represent the corresponding kernel density estimates. Mean values and standard deviations are indicated. Scale bars are 5 nm. False‐color height scales are shown in the corresponding panels and span 5 nm (a, b).

To provide a single‐particle perspective, CC_1 and CC_2 were imaged by AFM under liquid conditions on freshly cleaved highly ordered pyrolytic graphite (HOPG). Representative images of individual particles (Figure 4a,b) reveal clear differences in overall shape between the two constructs. The CC_1 frequently displayed elongated, anisotropic outlines, consistent with the more extended configuration inferred from SAXS. In contrast, CC_2 appeared more circular or mildly ellipsoidal, reflecting a compact organization. In several cases, individual subunits were also discernible, typically three to four per particle, in agreement with the expected architectures of both complexes. In a few complexes, a fifth subunit was also visible (not annotated), which may correspond to the CBM of the *At*Cel9R domain, consistent with previous AFM studies reporting distinguishable CBM and catalytic cores in related cellulases under similar imaging conditions (Eibinger et al., 2017). However, no attempt was made to assign these visible subunits to specific cellulase domains or Jo‐In components. These observations highlight that solution structures are preserved upon surface adsorption, at least on HOPG, directly revealing the structural variability between the two complexes.

Beyond visual inspection, quantitative analysis was performed to characterize particle size and shape. Maximal Feret diameters (equal to *D*_max_) were extracted for each particle, yielding distributions that distinguish CC_1 and CC_2 (Figure 4c,d). CC_1 displayed a slightly broader distribution with a higher average *D*_max_ of 190 ± 46 Å, whereas CC_2 showed a smaller average *D*_max_ of 178 ± 60 Å. Although absolute values are slightly larger than the SAXS‐derived *D*_max_ due to tip broadening (even with nominally sharp 1 nm probes (Wilson & Macpherson, 2009)), the relative differences between the two complexes appear to be retained. While this systematic broadening error should be kept in mind, the data nonetheless suggest that CC_1 adopts a more extended configuration, whereas CC_2 remains comparatively compact.

In addition to size, solidity, which is a measure of particle compactness defined as the ratio of the particle area to the area of its convex hull, was quantified (Figure 4e,f). The CC_1 exhibited a lower mean solidity (0.73 ± 0.11), consistent with elongated, anisotropic shapes, whereas CC_2 displayed higher mean solidity (0.81 ± 0.09), reflecting a more globular conformation. The solidity distribution of CC_1 is not significantly broader, but its center is slightly shifted toward lower values, with solidity values around 0.6 occurring notably more frequently. These experimental distributions are consistent with SAXS‐based interpretations, confirming the distinct topologies of the two assemblies. To further validate these findings, atomistic coordinate models derived from SAXS refinement and exported as PDB files were analyzed using an in‐house MATLAB workflow that simulated multiple surface‐bound orientations and generated projected AFM‐like particle footprints under simplified geometric assumptions. Although such PDB‐based models inherently represent a reduced conformational space compared with the full flexibility of biomolecular assemblies in solution, CC_1 and CC_2 consistently produced distinct solidity distributions across 2000 random orientations that were in good agreement with the experimental data (Figure S4). Notably, the same relative separation between the two complexes was retained in this simplified model, indicating that their differing shape descriptors arise primarily from intrinsic architectural differences. Together, these results support the notion that the AFM‐derived parameters capture genuine differences in molecular organization rather than being dominated by imaging artifacts or adsorption orientation alone.

AFM imaging shows that the morphological differences inferred from SAXS are retained upon adsorption to a hydrophobic surface. Both representative single‐particle images and statistical distributions obtained from several hundred particles confirm that CC_1 adopts more elongated shapes, whereas CC_2 remains more compact. Overall, AFM corroborated our SAXS‐dependent modeling workflow suggesting that this workflow could be applied to more complex enzyme assemblies.

### 2.6 SAXS analyses of trifunctional multi‐enzyme complexes

**FIGURE 5:**
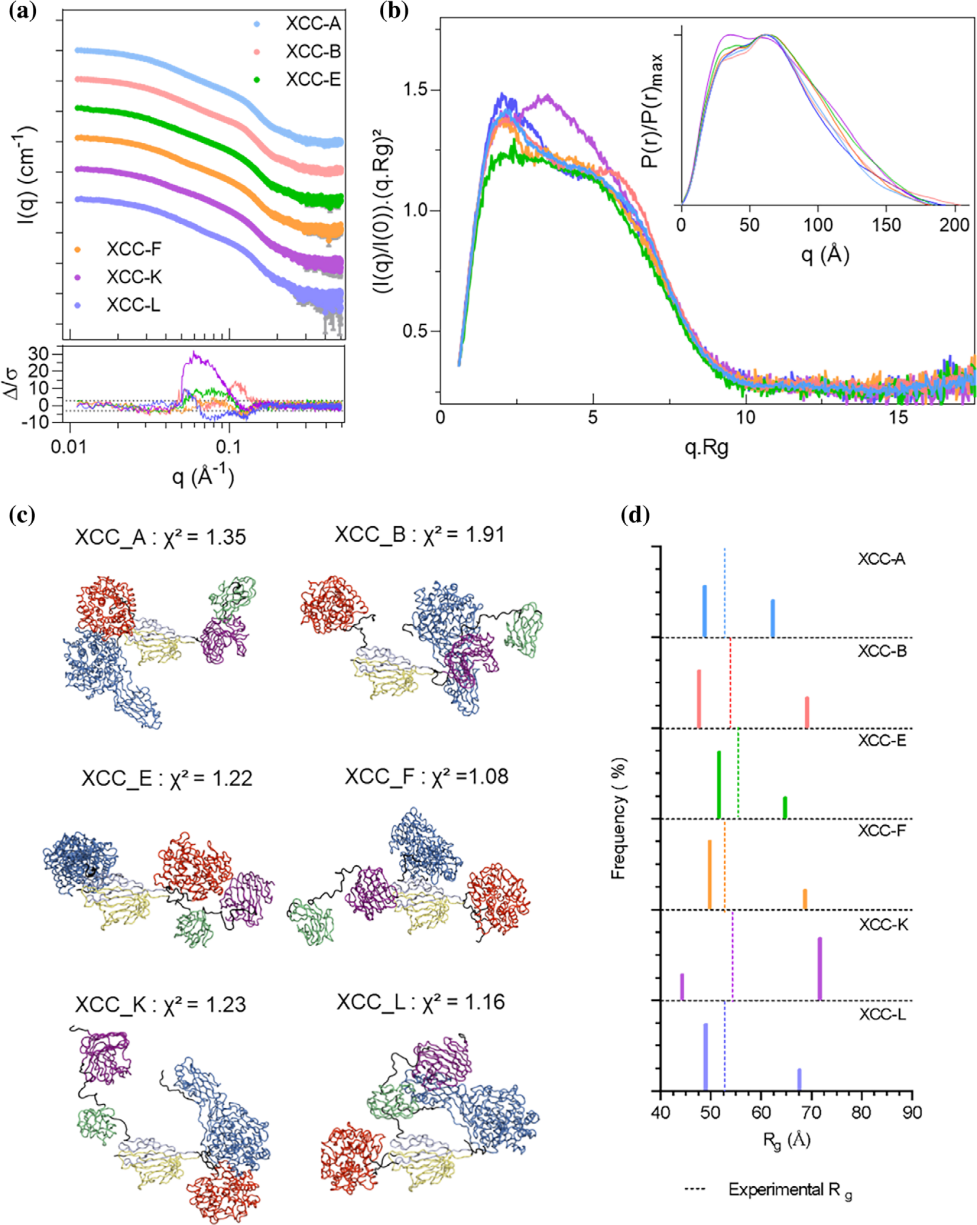
SAXS profile and modeling of the trifunctional assemblies. (a) Experimental SAXS profiles for the XCC_complexes, shown in distinct colors and vertically offset by powers of 10 for clarity. Residuals (bottom curves) are plotted relative to the XCC‐A scattering profile. (b) Dimensionless Kratky plots of the superimposed scattering data. Normalized pair‐distance distribution function *P*(*r*), scaled to their respective *P*(*r*)_max, is also shown in inset. (c) Most representative DADIMODO models for the six assemblies. Cel9R is shown in blue, Cel8A in red, XynCD (GH11) in violet, CBM6 in green and Jo–In in gray/yellow. (d) Bar plots of *R*_*g*_ values and relative abundances obtained from Minimal Ensemble Search (MES); dashed lines indicate experimentally obtained *R*_*g*_ values.

Since cellulosomes typically exceed two‐enzyme complexity, we extended the analysis to trifunctional assemblies combining the xylanase (*At*Xyn11A) with *At*Cel8A and *At*Cel9R. Six distinct complexes (XCC‐A to XCC‐L, Figure 1d) were generated by varying the fusion position with Jo–In. SAXS profiles are presented in Figure 5a and biophysical values are shown in Table 2. All complexes displayed similar low‐q scattering, indicating comparable overall dimensions and oligomeric states. Guinier analyses yielded *R*_*g*_ of 53.5 ± 0.9 Å, showing that addition of a third enzyme to the bifunctional complexes slightly increases size while reducing construct‐dependent differences in global dimensions. As for CC_1 and CC_2, normalized Kratky plots (Figure 5b) revealed consistent overall folding but distinct mid‐q behaviors (0.05–0.15 Å^−1^), characteristic of flexible multi‐domain systems. All complexes exhibited extended but folded conformations, as suggested by the return to baseline at high q.Rg. Differences in peak position and amplitude reflect variations in inter‐enzyme spacing within the complexes. Pair‐distance distribution functions *P(r)* (inset Figure 5b) were highly similar among the six assemblies, with *D*_max_ values ranging from 180 Å (XCC‐E, F, K) to 195 Å (XCC‐A, B, L), reflecting subtle differences in overall compactness. All *P*(*r*) functions exhibited two dominant maxima around 30 and 60 Å, and a third, weaker peak at 120 Å specifically in XCC‐F and XCC‐K, suggesting additional inter‐domain separations unique to those constructs. Overall, topological arrangements vary across enzyme complexes. While Jo–In core constrains compaction, subtle geometric differences yield distinct domain organization.

DADIMODO modeling was used to interpret the SAXS data at the atomistic level. Five independent optimization runs per complex produced satisfactory SAXS fits (*χ* ^2^ values listed in Table 2). Models superimposed on the Jo–In core (Figure S5) facilitate direct structural comparison. Overall, the assemblies exhibited maximum particle dimensions ranging from 161 Å for XCC‐L to 195 Å for XCC‐B (Table S5 and Figure S5). In most cases, the modeled structures appeared slightly more compact than suggested by experimental data, although the modeled *R*_*g*_ values remained close to the experimental measures. XCC‐K is the only complex marked with a *R*_*g*_ model higher than the experimental value. Despite an overall similarity in shape across complexes, differences in average organization were observed: XCC‐K adopts a more curved arrangement whereas XCC‐A, B, E, and L display more extended organizations and XCC‐F exhibits a distinct arrangement. The catalytic domains were generally well defined in the DADIMODO models, with most assemblies converging toward a consistent relative orientation across independent runs. Domains directly fused to the Jo–In core were systematically better resolved than distal domains, a trend particularly evident for the xylanase, whose CBM is connected via a 20‐residue linker. Direct CBM fusion to the Jo–In scaffold yielded less well‐defined catalytic domains, and vice versa, indicating that linker placement influences domain positioning and conformational sampling. Notably, enzyme spatial arrangement varied across complexes: for instance, in XCC‐A and XCC‐K the endoglucanase catalytic sites are in closer proximity, whereas in XCC‐L and XCC‐B they are further apart. The xylanase position remains more difficult to resolve due to intrinsic linker flexibility; however, the distance between its catalytic domain and CBM is consistent with the pair‐distance distribution functions shown in Figure 2c.

Given the increased flexibility of trifunctional assemblies, Minimal Ensemble Search (MES) analysis was performed. Consistent with previous DCs studies (Dorival et al., 2022), two conformations were sufficient to reproduce the scattering profiles. Increasing the number of conformers from one to two resulted in a substantial improvement of the fit, whereas adding additional conformers led only to marginal χ^2^ improvements, indicating a plateau and suggesting that two conformations capture the essential conformational variability without overfitting the data (Figure S7). Figure 5d shows the relative conformer population against *R*_*g*_, illustrating compactness distribution (all‐atom models in Figure S6). Because BILBO‐MD uses the MultiFoXS algorithm (Schneidman‐Duhovny et al., 2016), *χ* ^2^ values are calculated differently from CRYSOL‐based DADIMODO refinements and are therefore not directly comparable. Nevertheless, both approaches yield consistent trends, particularly in the intermediate q‐range. For all assemblies except XCC‐K, the predominant conformer exhibits an *R*_*g*_ smaller than the experimental value, indicating a dominant compact population within the ensemble, consistent with trends observed in the DADIMODO analysis. XCC‐F, XCC‐L, and XCC‐E are dominated by compact conformations (≥75%), whereas XCC‐A and XCC‐B display more balanced distributions between compact and extended states (52/48% and 65/35%, respectively) (Figure 5d). XCC‐K is the only complex predominantly sampling an extended conformation, representing nearly 70% of the ensemble. Consistent with DADIMODO modeling, which likewise predicts a more extended conformation, with modeled *R*_*g*_ exceeding those measured from Guinier analysis. These population ratios should be interpreted as indicative of relative conformational preferences rather than exact equilibrium distributions (Figure S6). Overall, the MES analysis supports a description of the assemblies in terms of dominant conformational states and average structural features (Figure 5c), rather than a single unique solution.

### 2.7 Determination of interdomain mean distances

**FIGURE 6:**
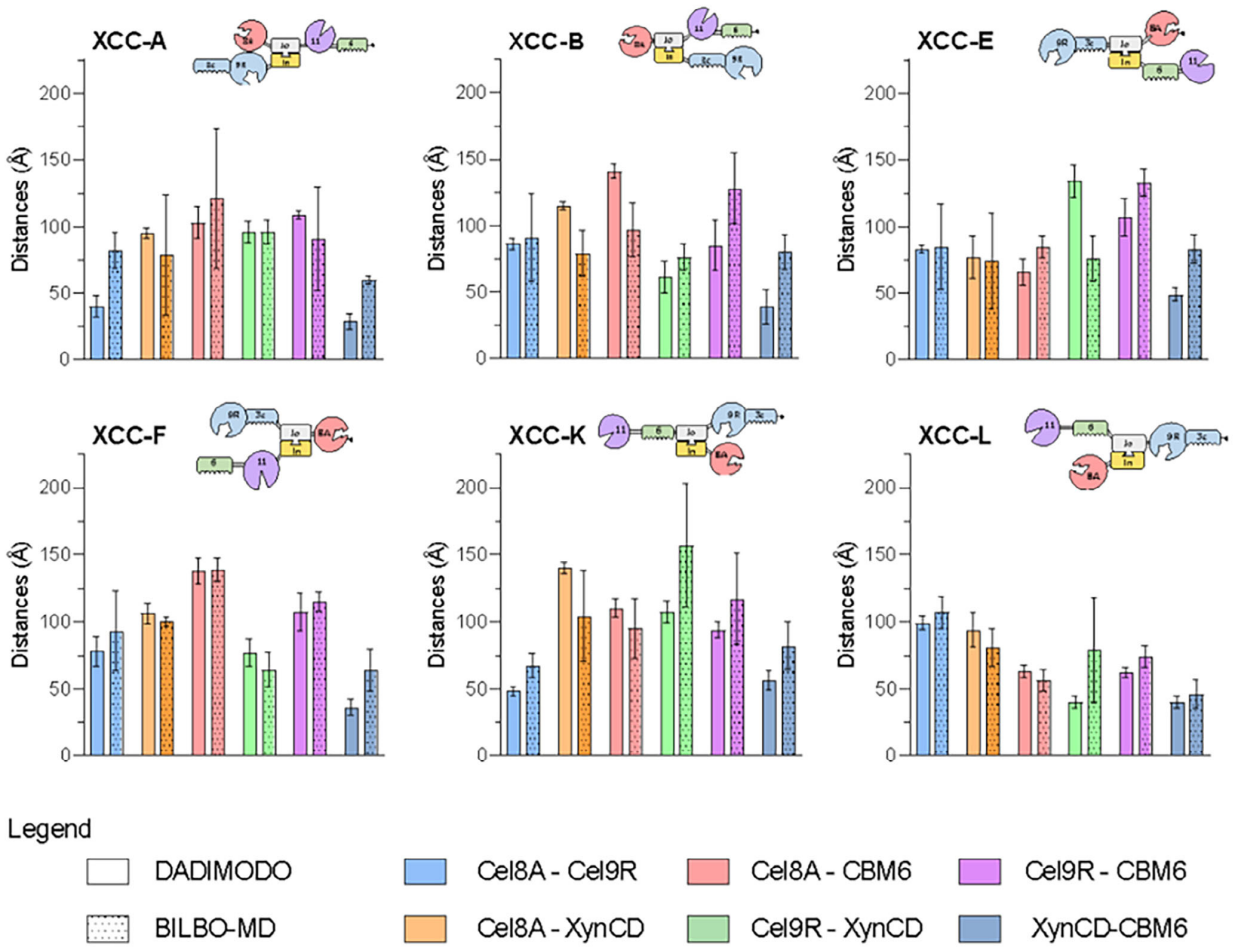
Bar plots showing the mean hypothetical distances between six domain pairs, each represented by a distinct color, with error bars indicating standard deviations calculated from five independent DADIMODO models or weighted mean for BILBO‐MD. The six panels correspond to the different XCC assemblies.

In order to quantify the differences between the complexes, the interdomain distances between catalytic amino acids of each domain were calculated (Table S4). Interdomain distances were extracted from models generated by either DADIMODO or BILBO‐MD, as illustrated in Figure 6. DADIMODO provides representative average conformation optimized to SAXS data, whereas BILBO‐MD samples weighted ensembles spanning compact and extended states. Consequently, BILBO‐MD‐derived distances display larger standard deviations, reflecting the broader conformational sampling. Despite these methodological differences, most interdomain distances are consistent between the two approaches, particularly for assemblies XCC‐F and XCC‐L, which exhibit similar distance profiles. In contrast, assemblies XCC‐A, XCC‐E, and XCC‐K show localized differences in specific interdomain separations. Specifically, XCC‐A exhibits a shorter distance between cellulase domains, while XCC‐E and XCC‐K showed reduced spacing between Cel9R and the catalytic domain of the xylanase. These variations likely arise from differences in domain connectivity, particularly in constructs where the xylanase domain is not directly fused to the Jo–In core. XCC‐B presents a unique distance pattern which can be rationalized by the presence of multiple conformational states identified in the MES analysis that are not fully captured by the averaged DADIMODO model. The xylanase–CBM distance serves as an internal reference: DADIMODO maintained a compact arrangement (~40 Å), close to what was observed in the autocorrelation function, whereas BILBO‐MD samples more extended conformations (~70 Å), contributing to the ensemble diversity. Overall, both approaches converge toward consistent trends in interdomain distances, indicating that the inferred spatial organization is robust with respect to the modeling strategy. These distances should be interpreted as representative average values derived from ensembles compatible with the SAXS data, rather than as uniquely defined geometric parameters.

## 3 DISCUSSION

**FIGURE 7:**
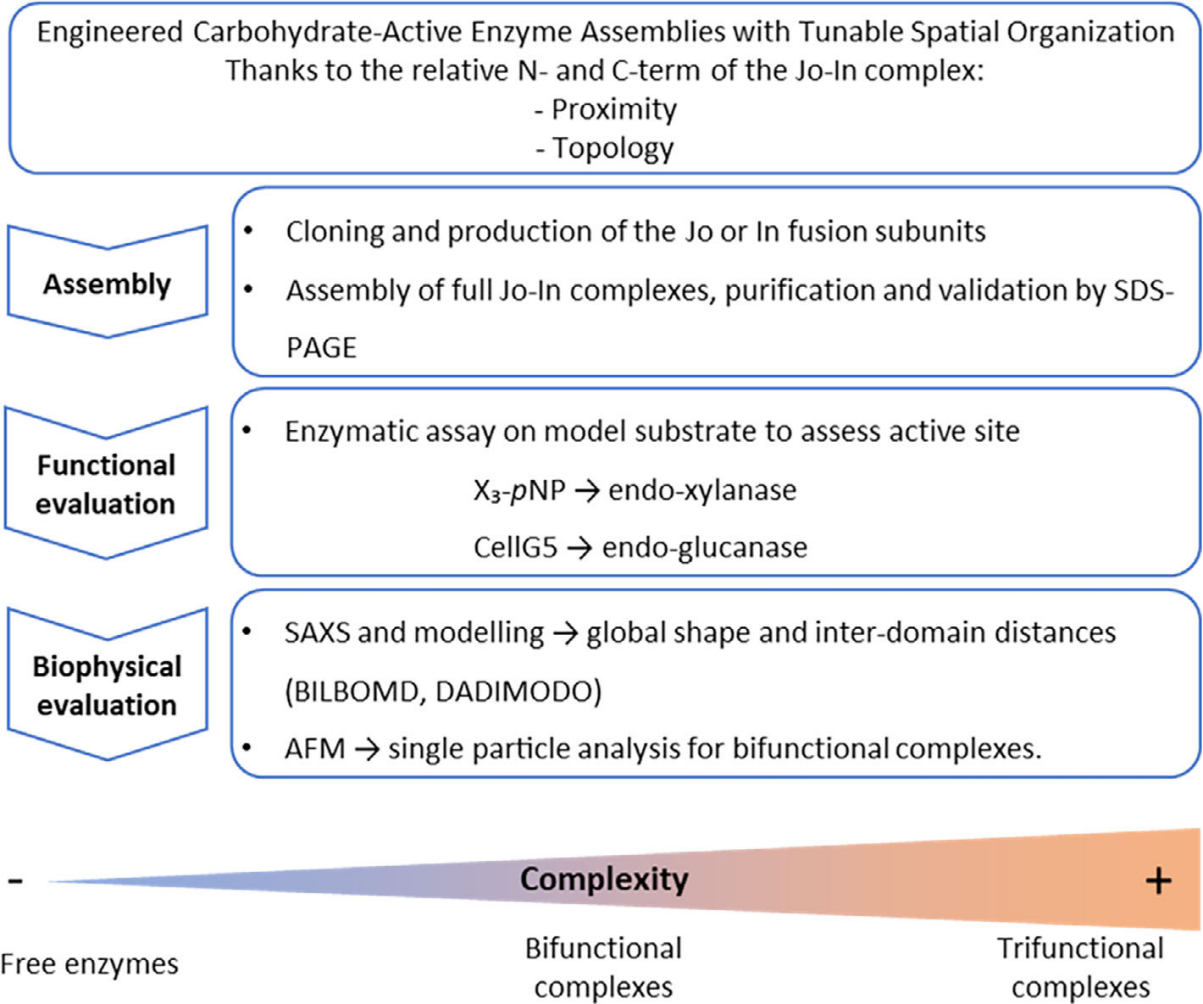
Workflow diagram illustrating the pipeline used in this study.

Natural cellulosomes exploit structural flexibility to hydrolyze insoluble plant cell wall substrates effectively (Artzi et al., 2017). However, their intrinsic conformational dynamics hampered our understanding of how spatial organization influences catalytic performance. Here, we designed multi‐enzyme complexes with controlled and distinct spatial arrangements using the Jo‐In welding system (Enjalbert et al., 2020). Our approach combines two cellulosomal endoglucanases *At*Cel8A and *At*Cel9R to form a bifunctional complex and an additional cellulosomal xylanase *At*Xyn11A to generate trifunctional complexes. By simply varying N or C‐terminal fusions to Jo or In, we access to tailor conformations (Figure 7). For each chimeric enzyme, the preservation of enzymatic activity is systematically assessed to ensure that structural constraints do not impair catalytic function. The spatial organization from free to multi‐enzyme architectures is systematically investigated using SAXS, and additionally using AFM for bifunctional complexes (Figure 7).

Our approach reveals that Jo‐In‐mediated assemblies adopt compact and constrained conformations. SAXS analysis of bifunctional complexes demonstrated that enzyme positioning directly influences overall topology and degree of compaction, with distinct scattering profiles corresponding to different spatial arrangements. Complementary AFM imaging validated these solution structures at the single‐particle level, confirming that CC_1 and CC_2 exhibit distinct shapes with differences in maximal Feret diameter and solidity (Figure S4) reflecting extended versus more compact architectures. Critically, AFM demonstrates that topologies observed in solution were preserved upon HOPG surface adsorption. Individual particle analysis further revealed conformational heterogeneity within each assembly type, indicating that Jo‐In‐linked complexes retain a degree of flexibility while maintaining defined topologies. Although AFM has been employed to visualize processive enzymes acting on crystalline cellulose (Eibinger et al., 2017) this rely on sustained directional movement along the substrate, since only *At*Cel9R exhibit processivity (Kuch et al., 2023) among the three enzymes in our complexes, AFM studies were not applicable here. Together, the integrated SAXS‐AFM strategy provided complementary structural insights: SAXS provided reliable ensemble‐averaged distances and overall topology, while AFM validated these models and reveals single‐molecule variability. These findings establish that while Jo‐In enforces global compaction, it allows geometric fine‐tuning of domain arrangements enabling rational design of multi‐enzyme assemblies with tailored architectures.

Atomistic modeling using DADIMODO and BILBO‐MD, combined with SAXS data and domain structures, converged toward consistent topologies with constrained interdomain distances, providing robust structural models for the trifunctional assemblies. Both approaches yielded higher calculated *R*_*g*_ values for XCC‐K, which displays the largest proportion of extended conformers, reflecting a conformational ensemble dominated by compact states but with a significant extended sub‐population. This may be rationalized by the architecture of XCC‐K, where the CBM is fused to the N‐terminus of Jo, potentially relaxing constraints on the Xyn11‐CM6 linker and favoring greater flexibility, in contrast to XCC‐A where fusion through the catalytic domain may restrict the conformational landscape. Together, the combined use of these computational and experimental approaches is well established and has proven reliable for structural characterization of designer cellulosomes (Dorival et al., 2022; Hammel et al., 2005; Molinier et al., 2011). While SAXS resolution inherently limits precise domain orientation assignments, extending the q‐range toward higher scattering angles would improve modeling constraints and refine domain localization. Moreover, combining molecular dynamics simulations with SAXS‐guided ensemble refinement could provide the most accurate description of these flexible assemblies (Pesce & Lindorff‐Larsen, 2021). Ultimately, high‐resolution cryo‐EM offers a complementary approach to validate SAXS‐derived structural models of these multi‐enzyme assemblies in solution. However, in the case of flexible proteins, it will likely require further methodological advances. Unlike dynamic cohesin–dockerin cellulosomes (Hammel et al., 2005; Molinier et al., 2011), Jo‐In forms a rigid covalent isopeptide bond (Bonnet et al., 2017; Cox et al., 2022) that reproducibly position enzymes, yielding compact and architecturally defined trifunctional assemblies. This structural rigidity is directly reflected in the biophysical parameters measured by SAXS: our trifunctional 180 kDa Jo‐In assemblies exhibit *R*_*g*_ ≈55 Å and *D*_max_ ≈190 Å, markedly more compact than a previously reported trifunctional designer cellulosome of approximately 300 kDa, which displays *R*_*g*_ ≈91 Å and *D*_max_ ≈305 Å. Although the two systems differ substantially in molecular weight, it is worth noting that the trifunctional DC harbors two identical enzymes out of three as in XCC (Cel8A and Cel9R) (Dorival et al., 2022). As highlighted by Dorival et al., local architectural features such as tightly tethered accessory modules or CBM insertions may further influence its hydrodynamic envelope and solution scattering profile. Within this context, the overall increased compactness of the Jo‐In assemblies most likely reflects the isopeptide‐bonded Jo‐In core acting as a rigid structural anchor that reduces the accessible conformational space and constrains the maximum interdomain distances achievable in solution. The ability to produce multi‐enzyme complexes with defined and reproducible topologies opens a direct path toward investigating how interdomain distances and the relative spatial arrangement of catalytic domains influence synergistic activity on insoluble substrates (Molinier et al., 2011; Moraïs et al., 2010). This is particularly timely given emerging structural data on the nanoscale organization of polysaccharides within the plant cell wall. Recent solid‐state NMR studies have begun to define the relative distances and arrangements of different polysaccharide classes (cellulose, hemicellulose, and pectins) within the native matrix (Addison et al., 2024; Dupree et al., 2015), providing a structural framework against which enzymatic assemblies can now be rationally designed. The availability of Jo‐In assemblies with distinct, controlled interdomain conformations therefore creates a unique experimental platform to test whether matching the spatial topology of a multi‐enzyme complex to the supramolecular organization of its substrate (plant cell wall) enhances catalytic performance—a hypothesis that has remained difficult to address rigorously with architecturally heterogeneous cellulosome systems. In this regard, the two approaches are rather complementary: cellulosomes provide natural diversity and adaptability, while Jo‐In assemblies could offer the geometric precision required to establish structure–activity relationships at the molecular level.

This work establishes genuine architectural rules for engineering multi‐enzyme systems that constitute a keystone to investigate the relationship between enzyme organization and activity. Gaining a deeper understanding of how synthetic assemblies interact with insoluble substrates including potential diffusion limitations, conformational constraints, and the dynamics of processive enzyme interactions will be critical for elucidating the role of spatial organization in modulating catalytic performance within multi‐enzyme systems. Jo‐In‐based assemblies offer a promising platform for both fundamental studies of enzyme cooperation and the rational design of biocatalytic systems. Their ability to form well‐defined complexes with controlled topology, combined with the flexibility observed at the single‐molecule level, provides a powerful framework for engineering optimized multi‐enzyme systems involved not only in insoluble substrate degradation but in many critical biological processes.

## 4 MATERIALS AND METHODS

### 4.1 Plasmid production

*At*Cel9R, *At*Cel8A, and *At*Xyn11A from *A. thermocellus* (formerly termed *C. thermocellum, Ruminiclostridium thermocellum*, and *Hungateiclostridium thermocellum*) and Jo‐In from *Streptococcus pneumoniae* RrgA open reading frames and corresponding sequences are shown in Table S1. Each gene was PCR‐amplified using Q5 Master Mix (NEB) and primers (Table S2), including *BsaI* recognition sites (GGTCTC) with 4‐nucleotide overhangs. PCR amplification followed standard cycling conditions (98°C 3 min; 29 cycles of 98°C 15 s, 60°C 15 s, and 72°C 10 s; final extension 72°C for 3 min). The pET28 was similarly amplified to incorporate BsaI sites. All purified fragments were assembled using Golden Gate cloning with Golden Gate Bridge Master Mix, supplemented with 20 U/reaction BsaV2‐HF (20 cycles of 37°C for 3 min and 16°C for 2 min, followed by 60°C for 5 min). Colonies were selected for plasmid propagation and extraction, and verified by Sanger sequencing.

### 4.2 Enzyme production

Recombinant enzymes were expressed in *E. coli* BL21(DE3) with C‐terminal His₆ tags (except untagged In‐chimeras). *At*Cel9R constructs used ZYP‐5052 autoinduction media (100 μg/mL kanamycin, 25°C, 24 h). Other enzymes were grown in LB with kanamycin to A₆₀₀ ≈0.6, induced with 1 mM IPTG, and cultured for 4 h at 37°C. Cells were harvested (5000×*g*, 15 min), resuspended at A₆₀₀ ≈40–80 in buffer (50 mM Tris–HCl, 300 mM NaCl, pH 8.0, protease inhibitors), and frozen at −80°C.

### 4.3 Multi‐enzyme assemblies

Expression of soluble chimeric enzymes containing Jo or In was rapidly assessed by lysing 1 mL aliquot of cells (1 min, speed, Fastprep, MP Biomedicals), loading cell free extract on SDS‐PAGE (precast any kd, Bio‐rad) and evaluating using Image LabTM Software (version 6.0.1 build 31, Bio‐rad). Multi‐enzyme assemblies were obtained by mixing cell free extracts containing both untagged In chimeric proteins in molar excess with their cell free extract of His₆‐tagged Jo chimeric partners, and the mixtures were incubated at room temperature (20–23°C) for 1.5 h under mild agitation (800 rpm, Thermomix, Eppendorf). Samples were heated at 50°C for 30 min, and recovered as the respective soluble fraction by centrifugation at 50,000×*g* for 30 min, 4°C.

### 4.4 Protein purification

Cell extracts were prepared by sonication (2.5 min, pulsed 1 min 5 kJ) and centrifugation (50,000×*g*, 30 min). His₆‐tagged proteins were purified by IMAC on cobalt resin (Takara), eluted with 100 mM imidazole, and desalted into 50 mM Tris–HCl, 150 mM NaCl, pH 8.0 with protease inhibitors (PD‐10 columns, Cytiva). Multi‐enzyme assemblies used nickel resin. SEC polishing (Superdex 200 10/300 GL, 1 mL/min) was performed when needed to reach >95% purity. Purity was confirmed by SDS‐PAGE (NuPAGE 3–8%) (Figure S8). Protein concentrations were determined by measuring absorbance at 280 nm A_2_₈₀ using calculated extinction coefficients (ExPASy ProtParam).

### 4.5 Enzymatic activities

*At*Xyn11A (20 nM final) was assayed with 2.5 mM of in house synthesized 4‐nitrophenyl‐β‐d‐xylotrioside, *p*NP‐X3 (Qiu & Fairbanks, 2020) in 50 mM sodium phosphate, pH 7, 0.1% BSA for 20 min at 50°C. Released 4‐nitrophenolate (A₄₀₁) was monitored continuously using a Cary 3500 Agilent Technologies at 20°C and quantified by *p*NP standards. One unit = 1 μmol pNP/min.

*At*Cel9R and *At*Cel8A activities were measured with the CellG5 kit (Megazyme) following the manufacturer's instructions. Briefly, reactions (20 nM enzyme in 100 μL of 50 mM acetate, pH 6, 0.1% BSA, and 4,6‐O‐(3‐ketobutylidene)‐4‐nitrophenyl‐β‐D‐cellopentaoside (BPNPG5) as substrate prewarmed to 50°C) ran for 10 min at 50°C, then were quenched with 800 μL of 2% Tris, pH 11. Absorbance was measured against *p*‐nitrophenol standards (0–500 μM). All assays were performed in triplicate.

### 4.6 SAXS data collection and primary analysis

SAXS measurements were performed on a XEUSS 2.0 system (Xenocs, France) bench SAXS with a Genix3D copper source (λ = 1.54 Å, 8 keV) delivering approximately 3 × 10^7^ photons·s^−1^ in a focused beam of about 500 × 500 μm^2^. Proteins (10–15 mg/mL; concentrated using Vivaspin filters) were analyzed under static measurement (40 μL, 18°C, vacuum). Data were collected on a Pilatus 1 M detector (1.216 m distance, *q* = 0.005–0.5 Å^−1^), averaging ≥12 exposures (600 s each).

Prior to SAXS measurements, samples were subjected to SEC (Superdex 200 10/300 GL). The peak apex was directly injected for initial SAXS analysis (40 μL). The remaining peak fractions were subsequently concentrated and injected to improve the signal‐to‐noise ratio at higher q. Scattering profiles from both injections (apex and concentrated fractions) were merged over a common q‐range where consistent overlap was observed, indicating minimal interparticle interactions.

Data quality was assessed by ensuring linear Guinier regions (*q*.*R*_*g*_ <1.3), confirming the absence of aggregation and the reliability of low‐q scattering (see Guinier plots, Figure S9). Buffer subtraction yielded scattering intensities *I*(*q*), and data reduction, including radial averaging and normalization, was performed using XSACT (Xenocs) following standard procedures.

Data deposition details: SAXS data collected at low and high concentration and merged were deposited in SAXSBDB (Kikhney et al., 2020) for each free enzyme and complexes. SASDYR7 (*At*Cel9R); (*At*Cel8A) SASDYT7 (*At*Xyn11A); SASDYU7 CC_1 (*At*Cel8A_Jo:In‐*At*Cel9R); SASDYV7 (CC_2 Jo_*At*Cel8A:In‐*At*Cel9R); SASDYW7 (XCC_A *At*Cel8A‐Jo‐*At*Xyn11A:In‐*At*Cel9R); SASDYX7 (XCC_B *At*Cel8A‐Jo‐*At*Xyn11A:*At*Cel9R‐In); SASDYY7 (XCC_E *At*Cel9R‐Jo‐*At*Cel8A:*At*Xyn11A_In); SASDYZ7 (XCC_F *At*Cel9R‐Jo‐*At*Cel8A:In‐*At*Xyn11A); SASDY28 (XCC_K *At*Xyn11A‐Jo‐*At*Cel9R:*At*Cel8A‐In); SASDY38 (XCC_L *At*Xyn11A‐Jo‐*At*Cel9R:In‐*At*Cel8A).

### 4.7 Modeling

Guinier analysis determined radius of gyration (*R*_*g*_), while indirect Fourier transformation provided pair‐distance distribution *P*(*r*), *D*_max_, and autocorrelation functions using PRIMUS ATSAS suite (Manalastas‐Cantos et al., 2021) and BioXTAS RAW 2 (Hopkins, 2024). Molecular weights were estimated from I(0)‐based methods.

Ab initio reconstructions used GASBOR (Svergun et al., 2001) (ATSAS online), generating 3 or 5 independent models (for free enzymes or complexes) and subsequently aligned and averaged using DAMAVER (Volkov & Svergun, 2003) to obtain the most representative shape envelope and the normalized discrepancy deviation (NSD) across the models.

For atomistic modeling, missing linker were reconstructed using MODELER 10.5 (Webb & Sali, 2016) based on available X‐ray structures (PDB: 1KWF, 7UNP, 1UXX, and 5MKC for Cel8A, Cel9R, CBM6, and Jo‐In, respectively) and AlphaFold predictions (AF‐O52780‐F1 for Xyn11A). All‐atom best 3D models (lowest DOPE scores) were refined using DADIMODO (https://dadimodo.synchrotron-soleil.fr), and generated models were aligned to obtain the most representative conformer. Five independent refined models were aligned to obtain the most representative conformer (Rudenko et al., 2019). To assess conformational flexibility, BILBO‐MD (https://bilbomd.bl1231.als.lbl.gov/) (Pelikan et al., 2009) was used to obtain the Minimal Ensemble Search (MES). 7200 models were generated (*R*_*g*_ range between 45 and 75 Å) and combined using MultiFoXs (Schneidman‐Duhovny et al., 2016) to produce the MES. Table S3 show the assignment of flexible residues.

### 4.8 AFM data collection and analysis

AFM measurements were performed using Dimension FastScan Bio™, (Bruker, MA) controlled via a Nanoscope V controller (Bruker) with NanoScope 9.2 software. Measurements used tapping mode in liquid at room temperature with FastScan DSS probes (Bruker; 110 kHz, 0.25 N/m, 1 nm tip radius).

Freshly cleaved highly ordered pyrolytic graphite (HOPG; grade I, SPI Supplies, PA) served as substrate. Immediately after cleavage, 200 μL of 50 mM MOPS pH 6.5 was applied to the surface. Once stable scanning was established, 20 μL of enzyme (200 μg/mL) was introduced and images were recorded at 1 × 1 μm^2^ (1024 × 1024 pixels, ≤1 nm/pixel).

Data were analyzed in Gwyddion, a free AFM analysis software (Nečas & Klapetek, 2012) with zero‐ or first‐order flattening. Images were split in smaller regions, for example, when an HOPG step edge resulted in two overlapping terraces. Height‐thresholded particles were converted to binary masks and exported. Exclusion criteria: area >300 pixels (aggregates), <75 pixels (debris), image borders, or *D*_max_ >30 pixels (unresolved oligomers).

Enzyme particles were first identified using a height threshold, after which the image was converted into a binary mask and exported as a .txt file. During subsequent processing, particles were excluded if their projected area >300 pixels (aggregates), <75 pixels (debris), or if located at the borders of the recorded image. The resulting .txt file was then imported into in‐house MATLAB (R2023b) routines to determine for each particle maximum Feret diameter (*D*_max_) and solidity (particle area/convex hull area). Particles exhibiting an apparent maximum Feret diameter larger than 30 pixels were also excluded, as such dimensions are physically implausible for the constructs studied and most likely reflect unresolved particle oligomers.

Simulated AFM projections were generated from SAXS‐derived PDB models using an in‐house MATLAB script. Atomic coordinates were centered and aligned along their principal axes prior to analysis. Multiple surface‐bound orientations were then sampled by applying random rigid‐body rotations with constrained tilt angles (up to 45°) relative to the surface normal, followed by two‐dimensional top‐view projection (Figure S4). To approximate finite AFM tip broadening, the resulting binary particle footprints were first slightly eroded and subsequently dilated using a circular structuring element corresponding to the nominal tip radius. Particle solidity was calculated from the processed projections and used as a reference for comparison with experimental AFM data.

## AUTHOR CONTRIBUTIONS

**Manuel Eibinger:** Software; formal analysis; writing – review and editing; writing – original draft; conceptualization; methodology; investigation; validation; visualization. **Jeremy Esque:** Software; validation; writing – review and editing; methodology; supervision; formal analysis. **Iker Pardo Larrabeiti:** Methodology; investigation; formal analysis; visualization; writing – original draft; writing – review and editing; conceptualization; software; validation. **Bernd Nidetzky:** Conceptualization; methodology; writing – original draft; writing – review and editing; validation; supervision. **Claire Dumon:** Conceptualization; writing – original draft; writing – review and editing; funding acquisition; validation; supervision; investigation; visualization; methodology; formal analysis. **Cédric Y. Montanier:** Conceptualization; investigation; funding acquisition; writing – original draft; writing – review and editing; visualization; methodology; validation; project administration; supervision; formal analysis. **Sébastien Fort:** Supervision; writing – review and editing; methodology. **Stéphanie Pradeau:** Conceptualization; methodology; validation. **Pierre Roblin:** Methodology; validation; software; formal analysis; conceptualization; writing – original draft; writing – review and editing; supervision; funding acquisition. **Itzhak Mizrahi:** Supervision; validation; resources. **Sarah Moraïs:** Conceptualization; methodology; validation; writing – review and editing; resources. **Edward A. Bayer:** Conceptualization; writing – review and editing; validation; resources; supervision.

## Supporting information


**TABLE S1.** Sequences of the Free enzymes and Jo‐In amino‐acid sequences.
**TABLE S2.** Primers used in this study.
**TABLE S3.** Flexible residues assignment.
**TABLE S4.** Amino acid involved in catalysis or recognition used to estimate interdomain distances.
**TABLE S5.** SASDB deposition table.
**DATA S1.** Supplementary material consists of enzyme sequences, SDS‐Page, and AFM, modeling of SAXS with DADIMODO and BILBO MD.

**FIGURE S1.** (a) Ab initio reconstructions of *At*Xyn11A generated using GASBOR and the corresponding NSD. (b) Five independent models of *At*Xyn11A superimposed into the catalytic domain.
**FIGURE S2.** Five independent models of the CC_1 (a) or CC_2 (b) assemblies generated with DADIMODO, shown as Cα ribbon representations superimposed on the JoIn core along with the corresponding χ^2^ values are reported in the left panel. The Cα NSD matrix is shown in the right panel. (c) Distances of 5 models displayed with mean and SD as displayed in Figure 4.
**FIGURE S3.** Ab initio reconstructions generated using GASBOR. Panels show five independent GASBOR models and the most representative envelope for CC_1 (a) and CC_2 (b), together with the corresponding NSD values.
**FIGURE S4.** Representative AFM‐like top‐view projections of CC_1 (a) and CC_2 (b) for a subset of randomly sampled orientations. Each panel shows the theoretical projected footprint after rasterization and AFM‐tip dilation, illustrating the variation in surface coverage. (c): Histograms of solidity (ratio of object area to convex hull area) for 2000 randomly sampled orientations of each complex. CC_1 (blue) shows slightly lower solidity, reflecting a more elongated projected shape, whereas CC_2 (red) displays a higher solidity, indicating a more compact footprint.
**FIGURE S5.** All‐atom modeling of the XCC complexes using DADIMODO. Five independent models were generated and superimposed within the JoIn scaffold, with the most representative model shown in bold. For each complex, the residual corresponding to the most representative model is displayed.
**FIGURE S6.** Modeling of XCC complexes using BILBO‐MD reveals the coexistence of compact and elongated conformations within the ensemble. The relative population of each conformational state is indicated, together with the corresponding radii of gyration (*R*_*g*_) and maximum dimensions (*D*_max_).
**FIGURE S7.** χ^2^ values for MES fit in dependence to the number of selected conformers for the 6 XCC assemblies showing a net decrease of the χ^2^ after 2 conformers followed by a plateau when more conformers are integrated.
**FIGURE S8.** SDS‐Page of the purified complexes and the free enzymes.
**FIGURE S9.** Guinier analysis of all assemblies and free enzymes used in this study. The inset displays the corresponding residuals of the fit, indicating the quality of the Guinier approximation within the selected *q*‐range.

## Data Availability

The data that support the findings of this study are openly available in SASBDB at https://www.sasbdb.org/.
