# Supplementary material for: Integrative SAXS and AFM analysis of engineered carbohydrate‐active enzyme assemblies with tunable spatial organization
Source: Protein Sci. 2026 Jun 15;35(7):e70649. doi: 10.1002/pro.70649 (PMC13269677; doi:10.1002/pro.70649)
Supplement: Supplementary file 1 — TABLE S1. Sequences of the Free enzymes and Jo‐In amino‐acid sequences. TABLE S2. Primers used in this study. TABLE S3. Flexible residues assignment. TABLE S4. Amino acid involved in catalysis or recognition used to estimate interdomain distances. TABLE S5. SASDB deposition table. DATA S1. Supplementary material consists of enzyme sequences, SDS‐Page, and AFM, modeling of SAXS with DADIMODO and BILBO MD. [file PRO-35-e70649-s002.docx]

**SUPPLEMENTARY Tables**

**Table S1: Sequences of the Free enzymes and Jo-In amino-acid sequences**

**Table S2: Primers used in this study**

**Table S3: Flexible residues assignment**

**Table S4: Amino acid involved in catalysis or recognition used to estimate interdomain distances**

**Table S5: SASDB deposition table**

**Table S1 : Free enzymes used and JoIn amino acid sequences.** Bold amino acids are considered as flexible for all atom modeling

| **Gene** | **Enz** | **AA sequence** |
| --- | --- | --- |
| Clo1313_1960  Genbank : ABN51508.1 | C-term 6His  Cel8A | **MGSSNAGVPFNTK**YPYGPTSIADNQSEVTAMLKAEWEDWKSKRITSNGAGGYKRVQRDASTNYDTVSEGMGYGLLLAVCFNEQALFDDLYRYVKSHFNGNGLMHWHIDANNNVTSHDGGDGAATDADEDIALALIFADKLWGSSGAINYGQEARTLINNLYNHCVEHGSYVLKPGDRWGGSSVTNPSYFAPAWYKVYAQYTGDTRWNQVADKCYQIVEEVKKYNNGTGLVPDWCTASGTPASGQSYDYKYDATRYGWRTAVDYSWFGDQRAKANCDMLTKFFARDGAKGIVDGYTIQGSKISNNHNASFIGPVAAASMTGYDLNFAKELYRETVAVKDSEYYGYYGNSLRLLTLLYITGNFPNPL**SDLSGQASHHHHHH*** |
| Clo1313_1659  Genbank: ABN51814.1 | C-term 6His  Cel9R | **MGSSNAADY**NYGEALQKAIMFYEFQMSGKLPDNIRNNWRGDSCLGDGSDVGLDLTGGWFDAGDHVKFNLPMAYTATMLAWAVYEYKDALQKSGQLGYLMDQIKWASDYFIRCHPEKYVYYYQVGNGDMDHRWWVPAECIDVQAPRPSYKVDLSNPGSTVTAGTAAALAATALVFKDTDPAYAALCIRHAKELFDFAETTMSDKGYTAALNFYTSHSGWYDELSWAGAWIYLADGDETYLEKAEKYVDKWPIESQTTYIAYSWGHCWDDVHYGAALLLAKITNKSLYKEAIERHLDYWTVGFNGQRVRYTPKGLAHLTDWGVLRHATTTAFLACVYSDWSECPREKANIYIDFAKKQADYALGSSGRSYVVGFGVNPPQHPHHRTAHSSWCDSQKVPEYHRHVLYGALVGGPDASDAYVDDIGNYVTNEVACDYNAGFVGLLAKMYEKYGGNPIPNFMAIEEKTNEEIYVEATANSNNGVELKTYLYNKSGWPARVCDKLSFRYFMDLTEYVSAGYNPNDITVSIIYSAAPTAKISKPILYDASKNIYYCEIDLSGTKIFPGSNSDHQKETQFRIQPPAGAPWDNTNDFSYQGIKKNGEVVKEMPVYEDGVLIF**GVEPNGTGASHHHHHH*** |
| Clo1313_0521  Genbank:  ABN54169.1 | C-term 6His  Xyn11A | **MGSSNA**DVVITSNQTGTHGGYNFEYWKDTGNGTMVLKDGGAFSCEWSNINNILFRKGFKYDETKTHDQLGYITVTYSCNYQPNGNSYLGVYGWTSNPLVEYYIIESWGTWRPPGATPKGTITVDGGTYEIYETTRVNQPSIKGTATFQQYWSVRTSKRTSGTISVTEHFKAWERLGMKMGKMYEVALVVEGYQSSGKADVTSMTIT**VGNAPSTSSPPGPTPEPTPRSAFS**KIEAEEYNSLKSSTIQTIGTSDGGSGIGYIESGDYLVFNKINFGNGANSFKARVASGADTPTNIQLRLGSPTGTLIGTLTVASTGGWNNYEEKSCSITNTTGQHDLYLVFSGPVNIDYFIFD**SKGVNPASHHHHHH*** |
| RrgA | Jo | **SDQ**YPQTGTYPDVQTPYQIIKVDGSEKNGQHKALNPNPYERVIPEGTLSKRIYQVNNLDDNQYGIELTVSGKTVYE**QKD** |
| RrgA | In | TEKKSIENGTITDPMGELIDLQLGTDGRFDPADYTLTANDGSRLENGQAVGGPQNDGGLLKNAKVLYDTTEKRIRVTGLYLGTDEKVTLTYNVRLNDEFVSNKFYDTNGRTTLHPKEVEQNTVRDFPIPKIR**D** |

**Table S2: Primers used in this study.** BsaI site GGTCTC, bold = overhang, underline 5’gene

| **Primers** | **Sequence (nt)** |
| --- | --- |
| M1_For_Cel8A | ttGGTCTCt**AATG**cgggtgtgccttttaacacaaaatac |
| M1_Rev_Cel8A | ttGGTCTCt**ACCT**ttggccggaaaggtcactca |
| M3_For_Cel8A | ttGGTCTCt**GCTA**cggtgtgccttttaacacaaaatac |
| M3_Rev_Cel8A | ttGGTCTCt**AAGC**ttggccggaaaggtcactca |
| M1_For_Cel9R | ttGGTCTCt**AATG**cggcagactataactatggagaagc |
| M1_Rev_Cel9R | ttGGTCTCt**ACCT**accggtaccattgggttctac |
| M3_For_Cel9R | ttGGTCTCt**GCTA**cgcagactataactatggagaagc |
| M3_Rev_Cel9R | ttGGTCTCt**AAGC**accggtaccattgggttctac |
| M1_For_Xyn11A | ttGGTCTCt**AATG**cggatgtagtaattacgtcaaaccag |
| M1_Rev_Xyn11A | ttGGTCTCt**ACCT**aggatttacacctttcgagtcg |
| M3_For_Xyn11A | ttGGTCTCt**GCTA**cgatgtagtaattacgtcaaaccag |
| M3_Rev_Xyn11A | ttGGTCTCt**AAGC**aggatttacacctttcgagtcg |
| M2_For_Jo | ttGGTCTCt**AGGT**attctgaccagtatccacaaacag |
| M2_Rev_Jo | ttGGTCTCt**TAGC**catctttttgttcatacactgttttcc |
| M2_For_In | ttGGTCTCt**AGGT**atactgaaaagaaatcaattgagaatgg |
| M2_Rev_In | ttGGTCTCt**AGGT**ctactgaaaagaaatcaattgagaatgg |
| pET28_For | ttGGTCTCg**GCTT**cacaccaccaccaccaccact |
| pET28_Rev | ttGGTCTCa**CATT**gctgctgcccatggtatatc |

**Table S3: Flexible residues assignment**

| CC_1 | CC_2 |
| --- | --- |
| **MGSSNAGVPFNT**KYPYGPTSIADNQSEVTAMLKAEWEDWKSKRITSNGAGGYKRVQRDASTNYDTVSEGMGYGLLLAVCFNEQALFDDLYRYVKSHFNGNGLMHWHIDANNNVTSHDGGDGAATDADEDIALALIFADKLWGSSGAINYGQEARTLINNLYNHCVEHGSYVLKPGDRWGGSSVTNPSYFAPAWYKVYAQYTGDTRWNQVADKCYQIVEEVKKYNNGTGLVPDWCTASGTPASGQSYDYKYDATRYGWRTAVDYSWFGDQRAKANCDMLTKFFARDGAKGIVDGYTIQGSKISNNHNASFIGPVAAASMTGYDLNFAKELYRETVAVKDSEYYGYYGNSLRLLTLLYITGNFPNPL**SDLSGQRYSDQ**YPQTGTYPDVQTPYQIIKVDGSEKNGQHKALNPNPYERVIPEGTLSKRIYQVNNLDDNQYGIELTVSGKTVYE**QKDGYHHHHHH**:**MGSSRY**TEKKSIENGTITDPMGELIDLQLGTDGRFDPADYTLTANDGSRLENGQAVGGPQNDGGLLKNAKVLYDTTEKRIRVTGLYLGTDEKVTLTYNVRLNDEFVSNKFYDTNGRTTLHPKEVEQNTVRDFPIPKIR**DGYADY**NYGEALQKAIMFYEFQMSGKLPDNIRNNWRGDSCLGDGSDVGLDLTGGWFDAGDHVKFNLPMAYTATMLAWAVYEYKDALQKSGQLGYLMDQIKWASDYFIRCHPEKYVYYYQVGNGDMDHRWWVPAECIDVQAPRPSYKVDLSNPGSTVTAGTAAALAATALVFKDTDPAYAALCIRHAKELFDFAETTMSDKGYTAALNFYTSHSGWYDELSWAGAWIYLADGDETYLEKAEKYVDKWPIESQTTYIAYSWGHCWDDVHYGAALLLAKITNKSLYKEAIERHLDYWTVGFNGQRVRYTPKGLAHLTDWGVLRHATTTAFLACVYSDWSECPREKANIYIDFAKKQADYALGSSGRSYVVGFGVNPPQHPHHRTAHSSWCDSQKVPEYHRHVLYGALVGGPDASDAYVDDIGNYVTNEVACDYNAGFVGLLAKMYEKYGGNPIPNFMAIEEKTNEEIYVEATANSNNGVELKTYLYNKSGWPARVCDKLSFRYFMDLTEYVSAGYNPNDITVSIIYSAAPTAKISKPILYDASKNIYYCEIDLSGTKIFPGSNSDHQKETQFRIQPPAGAPWDNTNDFSYQGIKKNGEVVKEMPVYEDGVLIF**GVEPNGTGAS*** | **MGSSRYSDQ**YPQTGTYPDVQTPYQIIKVDGSEKNGQHKALNPNPYERVIPEGTLSKRIYQVNNLDDNQYGIELTVSGKTVYE**QKDGYGVPFNT**KYPYGPTSIADNQSEVTAMLKAEWEDWKSKRITSNGAGGYKRVQRDASTNYDTVSEGMGYGLLLAVCFNEQALFDDLYRYVKSHFNGNGLMHWHIDANNNVTSHDGGDGAATDADEDIALALIFADKLWGSSGAINYGQEARTLINNLYNHCVEHGSYVLKPGDRWGGSSVTNPSYFAPAWYKVYAQYTGDTRWNQVADKCYQIVEEVKKYNNGTGLVPDWCTASGTPASGQSYDYKYDATRYGWRTAVDYSWFGDQRAKANCDMLTKFFARDGAKGIVDGYTIQGSKISNNHNASFIGPVAAASMTGYDLNFAKELYRETVAVKDSEYYGYYGNSLRLLTLLYITGNFPNPL**SDLSGQASHHHHHH**:**MGSSRY**TEKKSIENGTITDPMGELIDLQLGTDGRFDPADYTLTANDGSRLENGQAVGGPQNDGGLLKNAKVLYDTTEKRIRVTGLYLGTDEKVTLTYNVRLNDEFVSNKFYDTNGRTTLHPKEVEQNTVRDFPIPKI**RDGYADY**NYGEALQKAIMFYEFQMSGKLPDNIRNNWRGDSCLGDGSDVGLDLTGGWFDAGDHVKFNLPMAYTATMLAWAVYEYKDALQKSGQLGYLMDQIKWASDYFIRCHPEKYVYYYQVGNGDMDHRWWVPAECIDVQAPRPSYKVDLSNPGSTVTAGTAAALAATALVFKDTDPAYAALCIRHAKELFDFAETTMSDKGYTAALNFYTSHSGWYDELSWAGAWIYLADGDETYLEKAEKYVDKWPIESQTTYIAYSWGHCWDDVHYGAALLLAKITNKSLYKEAIERHLDYWTVGFNGQRVRYTPKGLAHLTDWGVLRHATTTAFLACVYSDWSECPREKANIYIDFAKKQADYALGSSGRSYVVGFGVNPPQHPHHRTAHSSWCDSQKVPEYHRHVLYGALVGGPDASDAYVDDIGNYVTNEVACDYNAGFVGLLAKMYEKYGGNPIPNFMAIEEKTNEEIYVEATANSNNGVELKTYLYNKSGWPARVCDKLSFRYFMDLTEYVSAGYNPNDITVSIIYSAAPTAKISKPILYDASKNIYYCEIDLSGTKIFPGSNSDHQKETQFRIQPPAGAPWDNTNDFSYQGIKKNGEVVKEMPVYEDGVLIF**GVEPNGTGAS*** |

| XCC-A | XCC-B |
| --- | --- |
| **MGSSNAGVPFNTK**YPYGPTSIADNQSEVTAMLKAEWEDWKSKRITSNGAGGYKRVQRDAS  TNYDTVSEGMGYGLLLAVCFNEQALFDDLYRYVKSHFNGNGLMHWHIDANNNVTSHDGGD  GAATDADEDIALALIFADKLWGSSGAINYGQEARTLINNLYNHCVEHGSYVLKPGDRWGG  SSVTNPSYFAPAWYKVYAQYTGDTRWNQVADKCYQIVEEVKKYNNGTGLVPDWCTASGTP  ASGQSYDYKYDATRYGWRTAVDYSWFGDQRAKANCDMLTKFFARDGAKGIVDGYTIQGSK  ISNNHNASFIGPVAAASMTGYDLNFAKELYRETVAVKDSEYYGYYGNSLRLLTLLYITGN  FPNPL**SDLSGQRYSDQ**YPQTGTYPDVQTPYQIIKVDGSEKNGQHKALNPNPYERVIPEGT  LSKRIYQVNNLDDNQYGIELTVSGKTVYE**QKDGY**DVVITSNQTGTHGGYNFEYWKDTGNG  TMVLKDGGAFSCEWSNINNILFRKGFKYDETKTHDQLGYITVTYSCNYQPNGNSYLGVYG  WTSNPLVEYYIIESWGTWRPPGATPKGTITVDGGTYEIYETTRVNQPSIKGTATFQQYWS  VRTSKRTSGTISVTEHFKAWERLGMKMGKMYEVALVVEGYQSSGKADVTSMTIT**VGNAPS**  **TSSPPGPTPEPTPRSAFS**KIEAEEYNSLKSSTIQTIGTSDGGSGIGYIESGDYLVFNKIN  FGNGANSFKARVASGADTPTNIQLRLGSPTGTLIGTLTVASTGGWNNYEEKSCSITNTTG  QHDLYLVFSGPVNIDYFIFD**SKGVNPASHHHHHH:MGSSRY**TEKKSIENGTITDPMGELI  DLQLGTDGRFDPADYTLTANDGSRLENGQAVGGPQNDGGLLKNAKVLYDTTEKRIRVTGL  YLGTDEKVTLTYNVRLNDEFVSNKFYDTNGRTTLHPKEVEQNTVRDFPIPKIR**DGYADY**N  YGEALQKAIMFYEFQMSGKLPDNIRNNWRGDSCLGDGSDVGLDLTGGWFDAGDHVKFNLP  MAYTATMLAWAVYEYKDALQKSGQLGYLMDQIKWASDYFIRCHPEKYVYYYQVGNGDMDH  RWWVPAECIDVQAPRPSYKVDLSNPGSTVTAGTAAALAATALVFKDTDPAYAALCIRHAK  ELFDFAETTMSDKGYTAALNFYTSHSGWYDELSWAGAWIYLADGDETYLEKAEKYVDKWP  IESQTTYIAYSWGHCWDDVHYGAALLLAKITNKSLYKEAIERHLDYWTVGFNGQRVRYTP  KGLAHLTDWGVLRHATTTAFLACVYSDWSECPREKANIYIDFAKKQADYALGSSGRSYVV  GFGVNPPQHPHHRTAHSSWCDSQKVPEYHRHVLYGALVGGPDASDAYVDDIGNYVTNEVA  CDYNAGFVGLLAKMYEKYGGNPIPNFMAIEEKTNEEIYVEATANSNNGVELKTYLYNKSG  WPARVCDKLSFRYFMDLTEYVSAGYNPNDITVSIIYSAAPTAKISKPILYDASKNIYYCE  IDLSGTKIFPGSNSDHQKETQFRIQPPAGAPWDNTNDFSYQGIKKNGEVVKEMPVYEDGV  LIF**GVEPNGTGAS*** | **MGSSNAGVPFNTK**YPYGPTSIADNQSEVTAMLKAEWEDWKSKRITSNGAGGYKRVQRDAS  TNYDTVSEGMGYGLLLAVCFNEQALFDDLYRYVKSHFNGNGLMHWHIDANNNVTSHDGGD  GAATDADEDIALALIFADKLWGSSGAINYGQEARTLINNLYNHCVEHGSYVLKPGDRWGG  SSVTNPSYFAPAWYKVYAQYTGDTRWNQVADKCYQIVEEVKKYNNGTGLVPDWCTASGTP  ASGQSYDYKYDATRYGWRTAVDYSWFGDQRAKANCDMLTKFFARDGAKGIVDGYTIQGSK  ISNNHNASFIGPVAAASMTGYDLNFAKELYRETVAVKDSEYYGYYGNSLRLLTLLYITGN  FPNPL**SDLSGQRYSDQ**YPQTGTYPDVQTPYQIIKVDGSEKNGQHKALNPNPYERVIPEGT  LSKRIYQVNNLDDNQYGIELTVSGKTVYE**QKDGY**DVVITSNQTGTHGGYNFEYWKDTGNG  TMVLKDGGAFSCEWSNINNILFRKGFKYDETKTHDQLGYITVTYSCNYQPNGNSYLGVYG  WTSNPLVEYYIIESWGTWRPPGATPKGTITVDGGTYEIYETTRVNQPSIKGTATFQQYWS  VRTSKRTSGTISVTEHFKAWERLGMKMGKMYEVALVVEGYQSSGKADVTSMTIT**VGNAPS**  **TSSPPGPTPEPTPRSAFS**KIEAEEYNSLKSSTIQTIGTSDGGSGIGYIESGDYLVFNKIN  FGNGANSFKARVASGADTPTNIQLRLGSPTGTLIGTLTVASTGGWNNYEEKSCSITNTTG  QHDLYLVFSGPVNIDYFIFD**SKGVNPASHHHHHH:MGSSNAADY**NYGEALQKAIMFYEFQ  MSGKLPDNIRNNWRGDSCLGDGSDVGLDLTGGWFDAGDHVKFNLPMAYTATMLAWAVYEY  KDALQKSGQLGYLMDQIKWASDYFIRCHPEKYVYYYQVGNGDMDHRWWVPAECIDVQAPR  PSYKVDLSNPGSTVTAGTAAALAATALVFKDTDPAYAALCIRHAKELFDFAETTMSDKGY  TAALNFYTSHSGWYDELSWAGAWIYLADGDETYLEKAEKYVDKWPIESQTTYIAYSWGHC  WDDVHYGAALLLAKITNKSLYKEAIERHLDYWTVGFNGQRVRYTPKGLAHLTDWGVLRHA  TTTAFLACVYSDWSECPREKANIYIDFAKKQADYALGSSGRSYVVGFGVNPPQHPHHRTA  HSSWCDSQKVPEYHRHVLYGALVGGPDASDAYVDDIGNYVTNEVACDYNAGFVGLLAKMY  EKYGGNPIPNFMAIEEKTNEEIYVEATANSNNGVELKTYLYNKSGWPARVCDKLSFRYFM  DLTEYVSAGYNPNDITVSIIYSAAPTAKISKPILYDASKNIYYCEIDLSGTKIFPGSNSD  HQKETQFRIQPPAGAPWDNTNDFSYQGIKKNGEVVKEMPVYEDGVLIF**GVEPNGTGRY**TE  KKSIENGTITDPMGELIDLQLGTDGRFDPADYTLTANDGSRLENGQAVGGPQNDGGLLKN  AKVLYDTTEKRIRVTGLYLGTDEKVTLTYNVRLNDEFVSNKFYDTNGRTTLHPKEVEQNT  VRDFPIPKIR**DGY*** |

| XCC-E | XCC-F |
| --- | --- |
| **MGSSNAADY**NYGEALQKAIMFYEFQMSGKLPDNIRNNWRGDSCLGDGSDVGLDLTGGWFD AGDHVKFNLPMAYTATMLAWAVYEYKDALQKSGQLGYLMDQIKWASDYFIRCHPEKYVYY YQVGNGDMDHRWWVPAECIDVQAPRPSYKVDLSNPGSTVTAGTAAALAATALVFKDTDPA YAALCIRHAKELFDFAETTMSDKGYTAALNFYTSHSGWYDELSWAGAWIYLADGDETYLE KAEKYVDKWPIESQTTYIAYSWGHCWDDVHYGAALLLAKITNKSLYKEAIERHLDYWTVG FNGQRVRYTPKGLAHLTDWGVLRHATTTAFLACVYSDWSECPREKANIYIDFAKKQADYA LGSSGRSYVVGFGVNPPQHPHHRTAHSSWCDSQKVPEYHRHVLYGALVGGPDASDAYVDD IGNYVTNEVACDYNAGFVGLLAKMYEKYGGNPIPNFMAIEEKTNEEIYVEATANSNNGVE LKTYLYNKSGWPARVCDKLSFRYFMDLTEYVSAGYNPNDITVSIIYSAAPTAKISKPILY DASKNIYYCEIDLSGTKIFPGSNSDHQKETQFRIQPPAGAPWDNTNDFSYQGIKKNGEVV KEMPVYEDGV**LIFGVEPNGTGRYSDQ**YPQTGTYPDVQTPYQIIKVDGSEKNGQHKALNPN PYERVIPEGTLSKRIYQVNNLDDNQYGIELTVSGKTVYE**QKDGYGVPFNT**KYPYGPTSIA DNQSEVTAMLKAEWEDWKSKRITSNGAGGYKRVQRDASTNYDTVSEGMGYGLLLAVCFNE QALFDDLYRYVKSHFNGNGLMHWHIDANNNVTSHDGGDGAATDADEDIALALIFADKLWG SSGAINYGQEARTLINNLYNHCVEHGSYVLKPGDRWGGSSVTNPSYFAPAWYKVYAQYTG DTRWNQVADKCYQIVEEVKKYNNGTGLVPDWCTASGTPASGQSYDYKYDATRYGWRTAVD YSWFGDQRAKANCDMLTKFFARDGAKGIVDGYTIQGSKISNNHNASFIGPVAAASMTGYD LNFAKELYRETVAVKDSEYYGYYGNSLRLLTLLYITGNFPNPL**SDLSGQASHHHHHH:MG** **SSNA**DVVITSNQTGTHGGYNFEYWKDTGNGTMVLKDGGAFSCEWSNINNILFRKGFKYDE TKTHDQLGYITVTYSCNYQPNGNSYLGVYGWTSNPLVEYYIIESWGTWRPPGATPKGTIT VDGGTYEIYETTRVNQPSIKGTATFQQYWSVRTSKRTSGTISVTEHFKAWERLGMKMGKM YEVALVVEGYQSSGKADVTSMTIT**VGNAPSTSSPPGPTPEPTPRSAFS**KIEAEEYNSLKS STIQTIGTSDGGSGIGYIESGDYLVFNKINFGNGANSFKARVASGADTPTNIQLRLGSPT GTLIGTLTVASTGGWNNYEEKSCSITNTTGQHDLYLVFSGPVNIDYFIFD**SKGVNPRY**TE KKSIENGTITDPMGELIDLQLGTDGRFDPADYTLTANDGSRLENGQAVGGPQNDGGLLKN AKVLYDTTEKRIRVTGLYLGTDEKVTLTYNVRLNDEFVSNKFYDTNGRTTLHPKEVEQNT VRDFPIPKIR**DGY*** | **MGSSNAADY**NYGEALQKAIMFYEFQMSGKLPDNIRNNWRGDSCLGDGSDVGLDLTGGWFD AGDHVKFNLPMAYTATMLAWAVYEYKDALQKSGQLGYLMDQIKWASDYFIRCHPEKYVYY YQVGNGDMDHRWWVPAECIDVQAPRPSYKVDLSNPGSTVTAGTAAALAATALVFKDTDPA YAALCIRHAKELFDFAETTMSDKGYTAALNFYTSHSGWYDELSWAGAWIYLADGDETYLE KAEKYVDKWPIESQTTYIAYSWGHCWDDVHYGAALLLAKITNKSLYKEAIERHLDYWTVG FNGQRVRYTPKGLAHLTDWGVLRHATTTAFLACVYSDWSECPREKANIYIDFAKKQADYA LGSSGRSYVVGFGVNPPQHPHHRTAHSSWCDSQKVPEYHRHVLYGALVGGPDASDAYVDD IGNYVTNEVACDYNAGFVGLLAKMYEKYGGNPIPNFMAIEEKTNEEIYVEATANSNNGVE LKTYLYNKSGWPARVCDKLSFRYFMDLTEYVSAGYNPNDITVSIIYSAAPTAKISKPILY DASKNIYYCEIDLSGTKIFPGSNSDHQKETQFRIQPPAGAPWDNTNDFSYQGIKKNGEVV KEMPVYEDGV**LIFGVEPNGTGRYSDQ**YPQTGTYPDVQTPYQIIKVDGSEKNGQHKALNPN PYERVIPEGTLSKRIYQVNNLDDNQYGIELTVSGKTVYE**QKDGYGVPFNT**KYPYGPTSIA DNQSEVTAMLKAEWEDWKSKRITSNGAGGYKRVQRDASTNYDTVSEGMGYGLLLAVCFNE QALFDDLYRYVKSHFNGNGLMHWHIDANNNVTSHDGGDGAATDADEDIALALIFADKLWG SSGAINYGQEARTLINNLYNHCVEHGSYVLKPGDRWGGSSVTNPSYFAPAWYKVYAQYTG DTRWNQVADKCYQIVEEVKKYNNGTGLVPDWCTASGTPASGQSYDYKYDATRYGWRTAVD YSWFGDQRAKANCDMLTKFFARDGAKGIVDGYTIQGSKISNNHNASFIGPVAAASMTGYD LNFAKELYRETVAVKDSEYYGYYGNSLRLLTLLYITGNFPNPL**SDLSGQASHHHHH:MG**  **SSRY**TEKKSIENGTITDPMGELIDLQLGTDGRFDPADYTLTANDGSRLENGQAVGGPQND  GGLLKNAKVLYDTTEKRIRVTGLYLGTDEKVTLTYNVRLNDEFVSNKFYDTNGRTTLHPK  EVEQNTVRDFPIPKI**RDGY**DVVITSNQTGTHGGYNFEYWKDTGNGTMVLKDGGAFSCEWS  NINNILFRKGFKYDETKTHDQLGYITVTYSCNYQPNGNSYLGVYGWTSNPLVEYYIIESW  GTWRPPGATPKGTITVDGGTYEIYETTRVNQPSIKGTATFQQYWSVRTSKRTSGTISVTE  HFKAWERLGMKMGKMYEVALVVEGYQSSGKADVTSMTIT**VGNAPSTSSPPGPTPEPTPRS**  **AFS**KIEAEEYNSLKSSTIQTIGTSDGGSGIGYIESGDYLVFNKINFGNGANSFKARVASG  ADTPTNIQLRLGSPTGTLIGTLTVASTGGWNNYEEKSCSITNTTGQHDLYLVFSGPVNID  YFIFD**SKGVNPAS*** |

| XCC-K | XCC-L |
| --- | --- |
| **MGSSNA**DVVITSNQTGTHGGYNFEYWKDTGNGTMVLKDGGAFSCEWSNINNILFRKGFKY  DETKTHDQLGYITVTYSCNYQPNGNSYLGVYGWTSNPLVEYYIIESWGTWRPPGATPKGT  ITVDGGTYEIYETTRVNQPSIKGTATFQQYWSVRTSKRTSGTISVTEHFKAWERLGMKMG  KMYEVALVVEGYQSSGKADVTSMTIT**VGNAPSTSSPPGPTPEPTPRSAFS**KIEAEEYNSL  KSSTIQTIGTSDGGSGIGYIESGDYLVFNKINFGNGANSFKARVASGADTPTNIQLRLGS  PTGTLIGTLTVASTGGWNNYEEKSCSITNTTGQHDLYLVFSGPVNIDYFIFD**SKGVNPRY**  **SDQ**YPQTGTYPDVQTPYQIIKVDGSEKNGQHKALNPNPYERVIPEGTLSKRIYQVNNLDD  NQYGIELTVSGKTVYE**QKDGYADY**NYGEALQKAIMFYEFQMSGKLPDNIRNNWRGDSCLG  DGSDVGLDLTGGWFDAGDHVKFNLPMAYTATMLAWAVYEYKDALQKSGQLGYLMDQIKWA  SDYFIRCHPEKYVYYYQVGNGDMDHRWWVPAECIDVQAPRPSYKVDLSNPGSTVTAGTAA  ALAATALVFKDTDPAYAALCIRHAKELFDFAETTMSDKGYTAALNFYTSHSGWYDELSWA  GAWIYLADGDETYLEKAEKYVDKWPIESQTTYIAYSWGHCWDDVHYGAALLLAKITNKSL  YKEAIERHLDYWTVGFNGQRVRYTPKGLAHLTDWGVLRHATTTAFLACVYSDWSECPREK  ANIYIDFAKKQADYALGSSGRSYVVGFGVNPPQHPHHRTAHSSWCDSQKVPEYHRHVLYG  ALVGGPDASDAYVDDIGNYVTNEVACDYNAGFVGLLAKMYEKYGGNPIPNFMAIEEKTNE  EIYVEATANSNNGVELKTYLYNKSGWPARVCDKLSFRYFMDLTEYVSAGYNPNDITVSII  YSAAPTAKISKPILYDASKNIYYCEIDLSGTKIFPGSNSDHQKETQFRIQPPAGAPWDNT  NDFSYQGIKKNGEVVKEMPVYEDGV**LIFGVEPNGTGASHHHHHH:MGSSNAGVPFNT**KYP  YGPTSIADNQSEVTAMLKAEWEDWKSKRITSNGAGGYKRVQRDASTNYDTVSEGMGYGLL  LAVCFNEQALFDDLYRYVKSHFNGNGLMHWHIDANNNVTSHDGGDGAATDADEDIALALI  FADKLWGSSGAINYGQEARTLINNLYNHCVEHGSYVLKPGDRWGGSSVTNPSYFAPAWYK  VYAQYTGDTRWNQVADKCYQIVEEVKKYNNGTGLVPDWCTASGTPASGQSYDYKYDATRY  GWRTAVDYSWFGDQRAKANCDMLTKFFARDGAKGIVDGYTIQGSKISNNHNASFIGPVAA  ASMTGYDLNFAKELYRETVAVKDSEYYGYYGNSLRLLTLLYITGNFPNPL**SDLSGQRY**TE  KKSIENGTITDPMGELIDLQLGTDGRFDPADYTLTANDGSRLENGQAVGGPQNDGGLLKN  AKVLYDTTEKRIRVTGLYLGTDEKVTLTYNVRLNDEFVSNKFYDTNGRTTLHPKEVEQNT  VRDFPIPKIR**DGY*** | **MGSSNA**DVVITSNQTGTHGGYNFEYWKDTGNGTMVLKDGGAFSCEWSNINNILFRKGFKY  DETKTHDQLGYITVTYSCNYQPNGNSYLGVYGWTSNPLVEYYIIESWGTWRPPGATPKGT  ITVDGGTYEIYETTRVNQPSIKGTATFQQYWSVRTSKRTSGTISVTEHFKAWERLGMKMG  KMYEVALVVEGYQSSGKADVTSMTIT**VGNAPSTSSPPGPTPEPTPRSAFS**KIEAEEYNSL  KSSTIQTIGTSDGGSGIGYIESGDYLVFNKINFGNGANSFKARVASGADTPTNIQLRLGS  PTGTLIGTLTVASTGGWNNYEEKSCSITNTTGQHDLYLVFSGPVNIDYFIFD**SKGVNPRY**  **SDQ**YPQTGTYPDVQTPYQIIKVDGSEKNGQHKALNPNPYERVIPEGTLSKRIYQVNNLDD  NQYGIELTVSGKTVYE**QKDGYADY**NYGEALQKAIMFYEFQMSGKLPDNIRNNWRGDSCLG  DGSDVGLDLTGGWFDAGDHVKFNLPMAYTATMLAWAVYEYKDALQKSGQLGYLMDQIKWA  SDYFIRCHPEKYVYYYQVGNGDMDHRWWVPAECIDVQAPRPSYKVDLSNPGSTVTAGTAA  ALAATALVFKDTDPAYAALCIRHAKELFDFAETTMSDKGYTAALNFYTSHSGWYDELSWA  GAWIYLADGDETYLEKAEKYVDKWPIESQTTYIAYSWGHCWDDVHYGAALLLAKITNKSL  YKEAIERHLDYWTVGFNGQRVRYTPKGLAHLTDWGVLRHATTTAFLACVYSDWSECPREK  ANIYIDFAKKQADYALGSSGRSYVVGFGVNPPQHPHHRTAHSSWCDSQKVPEYHRHVLYG  ALVGGPDASDAYVDDIGNYVTNEVACDYNAGFVGLLAKMYEKYGGNPIPNFMAIEEKTNE  EIYVEATANSNNGVELKTYLYNKSGWPARVCDKLSFRYFMDLTEYVSAGYNPNDITVSII  YSAAPTAKISKPILYDASKNIYYCEIDLSGTKIFPGSNSDHQKETQFRIQPPAGAPWDNT  NDFSYQGIKKNGEVVKEMPVYEDGV**LIFGVEPNGTGASHHHHHH**/**MGSSRY**TEKKSIENG  TITDPMGELIDLQLGTDGRFDPADYTLTANDGSRLENGQAVGGPQNDGGLLKNAKVLYDT  TEKRIRVTGLYLGTDEKVTLTYNVRLNDEFVSNKFYDTNGRTTLHPKEVEQNTVRDFPIP  KIR**DGYGVPFNT**KYPYGPTSIADNQSEVTAMLKAEWEDWKSKRITSNGAGGYKRVQRDAS  TNYDTVSEGMGYGLLLAVCFNEQALFDDLYRYVKSHFNGNGLMHWHIDANNNVTSHDGGD  GAATDADEDIALALIFADKLWGSSGAINYGQEARTLINNLYNHCVEHGSYVLKPGDRWGG  SSVTNPSYFAPAWYKVYAQYTGDTRWNQVADKCYQIVEEVKKYNNGTGLVPDWCTASGTP  ASGQSYDYKYDATRYGWRTAVDYSWFGDQRAKANCDMLTKFFARDGAKGIVDGYTIQGSK  ISNNHNASFIGPVAAASMTGYDLNFAKELYRETVAVKDSEYYGYYGNSLRLLTLLYITGN  FPNPL**SDLSGQAS*** |

**Table S4: Amino acid involved in catalysis or recognition to estimate interdomain distances**

|  | Domains | | | | |
| --- | --- | --- | --- | --- | --- |
| Constructs | **Cel8A** | **Cel9R** | **JoIn** | **Xyn GH11** | **CBM6** |
| Cel8A | D125 | nd | nd | nd | nd |
| Cel9R | nd | E428 | nd | nd | nd |
| Xyn11A | nd | nd | nd | E212 | Y281 |
| CC_1 | D251 | E1023 | K423 | nd | nd |
| CC_2 | D332 | E1041 | K123 | nd | nd |
| XCC_A | D125 | E1377 | K423 | E548 | Y792 |
| XCC_B | D125 | E1242 | K423 | E548 | Y792 |
| XCC_E | D949 | E428 | K673 | E1267 | Y1336 |
| XCC_F | D949 | E428 | K673 | E1402 | Y1471 |
| XCC_K | D1321 | E863 | K410 | E190 | Y259 |
| XCC_L | D1456 | E863 | K410 | E190 | Y259 |

**Table S5 : SASDB deposition table**

|  |  | AtCel8A-CD | AtCel9R-CDCBM | AtXyn11A | CC_1 | CC_2 | XCC-A | XCC-B | XCC-E | XCC-F | XCC-K | XCC-L |  |
| --- | --- | --- | --- | --- | --- | --- | --- | --- | --- | --- | --- | --- | --- |
|  | | | | | | | | | | | | | |
|  | Temperature | 20 °C | | | | | | | | | | | |
|  | Solvent composition | 25 mM Tris - 150 mM NaCl pH 8 | | | | | | | | | | | |
|  | Sample concentration (mg/mL) - Post SEC fraction | 3 | 3 | 3 | 0,9 | 1,7 | 8,6 | 7,5 | 1,0 | 1,2 | 1,9 | 1,9 |  |
|  | Sample concentration (mg/mL) - Concentrated fraction | 15 | 15 | 36 | 12,4 | 20,4 | 33,6 | 42,0 | 15,4 | 14,7 | 11,6 | 17,8 |  |
| Structural parameters | | | | | | | | | | | | | |
| Guinier analysis | |  |  |  |  |  |  |  |  |  |  |  |  |
|  | I(0) (cm^-1^) | 0,00312 | 0,00623 | 0,00347 | 0,01230 | 0,01230 | 0,1306 | 0,1154 | 0,0134 | 0,0150 | 0,0289 | 0,0291 |  |
|  | Rg (Å) | 20,4 ± 0,2 | 30,8 ± 0,2 | 29,0 ± 0,1 | 46,6 ± 0,5 | 43,0 ± 0,2 | 53,6 ± 0,2 | 52,8± 0,2 | 54,74 ± 0,2 | 53,7 ± 0,4 | 55,0 ± 0,5 | 53,7 ± 0,5 |  |
|  | qRg range (Å^-1^) | 0,22-1,30 | 0,24-1,29 | 0,29 - 1,30 | 0,49-1,26 | 0,43 - 1,29 | 0,59 - 1,30 | 0,58 - 1,28 | 0,7 - 1,30 | 0,6 - 1,30 | 0,61 - 1,30 | 0,60 - 1,3 |  |
|  | Coefficient of correlation, R² | 0,97 | 0,98 | 0,99 | 0,99 | 0,99 | 0,99 | 0,99 | 0,99 | 0,99 | 0,99 | 0,99 |  |
| P(r) analysis (GNOM) | |  |  |  |  |  |  |  |  |  |  |  |  |
|  | I(0) (cm-1) | 0,00309 | 0,00612 | 0,00355 | 0,01230 | 0,01230 | 0,1330 | 0,1154 | 0,0134 | 0,0152 | 0,0289 | 0,0291 |  |
|  | Rg (Å) | 20,5 ± 0,3 | 30,3 ± 0,1 | 31,0 ± 0,1 | 50,1 ± 0,3 | 42,0 ± 0,1 | 56,5 ± 0,1 | 53,7 ± 0,1 | 56,1 ± 0,2 | 54,8 ± 0,2 | 56,1 ± 0,2 | 54,8 ± 0,3 |  |
|  | Dmax (Å) | 64 | 100 | 100 | 172 | 130 | 204 | 187 | 190 | 183 | 180 | 193 |  |
|  | q range (Å-1) | 0,01-0,5 | 0,0145 - 0,5 | 0,01-0,5 | 0,01-0,5 | 0,01-0,5 | 0,0111 - 0,5 | 0,0111 - 0,5 | 0,0134 - 0,5 | 0,0111 - 0,5 | 0,0111 - 0,5 | 0,0111 - 0,5 |  |
|  | χ² (Total Estimate) | 1,25 (0,87) | 1,19 (0,80) | 1,26 (0,72) | 1,23 (0,76) | 1,17 (0,84) | 1,20 (0,81) | 1,37 (0,87) | 1,22 (0,81) | 1,06 (0,88) | 1,14 (0,76) | 1,03 (0,84) |  |
| Molecular weight analysis | |  |  |  |  |  |  |  |  |  |  |  |  |
|  | Corrected Porod volume, Vp (Å^3^) | 45 600 | 79 900 | 50 400 | 172 000 | 166 000 | 233 000 | 234 000 | 233 000 | 244 000 | 230 000 | 243 000 |  |
|  | MW from Vp (kDa) | 38 | 66 | 42 | 142 | 138 | 194 | 195 | 194 | 202 | 191 | 202 |  |
|  | MW from volume of correlation, Vc (kDa) | 37 | 62 | 36 | 121 | 114 | 164 | 166 | 162 | 172 | 157 | 169 |  |
|  | MW from sequence (kDa) | 42 | 72 | 38 | 136 | 136 | 175 | 175 | 175 | 175 | 175 | 175 |  |
|  | Oligomeric state | monomer | monomer | monomer | monomer | monomer | monomer | monomer | monomer | monomer | monomer | monomer |  |
| Ab initio bead modelling | | | | | | | | | | | | | |
|  | q range for fitting (Å-1) | 0,01 - 0,5 | 0,01 - 0,5 | 0,01 - 0,5 | 0,01 - 0,5 | 0,01 - 0,5 | 0,01 - 0,5 | 0,01 - 0,5 | 0,01 - 0,5 | 0,01 - 0,5 | 0,01 - 0,5 | 0,01 - 0,5 |  |
|  | GASBOR |  |  |  |  |  |  |  |  |  |  |  |  |
|  | Number of models generated | 3 | 3 | 3 | 5 | 5 | N.D. | N.D. | N.D. | N.D. | N.D. | N.D. |  |
|  | NSD (standard deviation) | 0,82 ± 0,02 | 0,88 ± 0,01 | 1,01 ± 0,02 | 1,7 ± 0,2 | 1,6 ± 0,2 | N.D. | N.D. | N.D. | N.D. | N.D. | N.D. |  |
|  | X² range | 2,17 - 2,30 | 1,75-1,90 | 1,42 - 1,98 | 1,26-1,33 | 1,29-1,50 | N.D. | N.D. | N.D. | N.D. | N.D. | N.D. |  |
|  | X² most representative model | 2,17 | 1,75 | 1,42 | 1,29 | 1,45 | N.D. | N.D. | N.D. | N.D. | N.D. | N.D. |  |
| Atomistic modelling | | | | | | | | | | | | | |
|  | Missing sequence modelling | Modeller 3.0 | | | | | | | | | | | |
|  |  |  | | | | | | | | | | | |
|  | Three-dimensional representations | Pymol 3.5 | | | | | | | | | | | |
|  | Model construction |  |  |  |  |  |  | | | | | |  |
|  | Crystal structures / Alphafold model used | 1kwf | 7unp | AF-O52779-F1-v4 | 1kwf; 7unp;5MKC | |  | | | | | |  |
|  | q range for fitting (Å-1) | 0,01-0,5 | 0,01-0,5 | 0,01-0,5 | 0,01-0,5 | 0,01-0,5 | 0,01-0,5 | 0,01-0,5 | 0,01-0,5 | 0,01-0,5 | 0,01-0,5 | 0,01-0,5 |  |
|  | CRYSOL |  |  |  |  |  |  |  |  |  |  |  |  |
|  | PDB used | 1kwf | 7unp | N.D. | N.D. | N.D. | N.D. | N.D. | N.D. | N.D. | N.D. | N.D. |  |
|  | X² | 1,95 | 2,4 | N.D. | N.D. | N.D. | N.D. | N.D. | N.D. | N.D. | N.D. | N.D. |  |
|  | Bilbo-MD |  |  |  |  |  |  |  |  |  |  |  |  |
|  | Domain assignation |  |  |  |  |  |  |  |  |  |  |  |  |
|  | Models generated | N.D. | N.D. | N.D. | N.D. | N.D. | 4 800 | 4 800 | 4 800 | 4 800 | 4 800 | 4 800 |  |
|  | Rg range Å | N.D. | N.D. | N.D. | N.D. | N.D. | 43-78 | 43-78 | 43-78 | 43-78 | 43-78 | 43-78 |  |
|  | MES number of conformers (X² value) | N.D. | N.D. | N.D. | N.D. | N.D. | 2 (2,6) | 2 (2,82) | 2 (1,38) | 2 (1,32) | 2 (3,05) | 2 (2,41) |  |
|  | DADIMODO |  |  |  |  |  |  |  |  |  |  |  |  |
|  | Models generated | N.D. | N.D. | 5 | 5 | 5 | 5 | 5 | 5 | 5 | 5 | 5 |  |
|  | X² range | N.D. | N.D. | 1,23-2,6 | 1,61-2,96 | 1,14-1,61 | 1,47-1,92 | 1,35-1,56 | 1,08-1,33 | 1,09-1,19 | 1,13-1,24 | 1,20-1,60 |  |
|  | Most representative model X² | N.D. | N.D. | 1,23 | 1,60 | 1,17 | 1,91 | 1,14 | 1,22 | 1,08 | 1,23 | 1,16 |  |
