# Supplementary material for: Integrative SAXS and AFM analysis of engineered carbohydrate‐active enzyme assemblies with tunable spatial organization
Source: Protein Sci. 2026 Jun 15;35(7):e70649. doi: 10.1002/pro.70649 (PMC13269677; doi:10.1002/pro.70649)
Supplement: Supplementary file 2 — FIGURE S1. (a) Ab initio reconstructions of AtXyn11A generated using GASBOR and the corresponding NSD. (b) Five independent models of AtXyn11A superimposed into the catalytic domain. FIGURE S2. Five independent models of the CC_1 (a) or CC_2 (b) assemblies generated with DADIMODO, shown as Cα ribbon representations superimposed on the JoIn core along with the corresponding χ2 values are reported in the left panel. The Cα NSD matrix is shown in the right panel. (c) Distances of 5 models displayed with mean and SD as displayed in Figure 4. FIGURE S3. Ab initio reconstructions generated using GASBOR. Panels show five independent GASBOR models and the most representative envelope for CC_1 (a) and CC_2 (b), together with the corresponding NSD values. FIGURE S4. Representative AFM‐like top‐view projections of CC_1 (a) and CC_2 (b) for a subset of randomly sampled orientations. Each panel shows the theoretical projected footprint after rasterization and AFM‐tip dilation, illustrating the variation in surface coverage. (c): Histograms of solidity (ratio of object area to convex hull area) for 2000 randomly sampled orientations of each complex. CC_1 (blue) shows slightly lower solidity, reflecting a more elongated projected shape, whereas CC_2 (red) displays a higher solidity, indicating a more compact footprint. FIGURE S5. All‐atom modeling of the XCC complexes using DADIMODO. Five independent models were generated and superimposed within the JoIn scaffold, with the most representative model shown in bold. For each complex, the residual corresponding to the most representative model is displayed. FIGURE S6. Modeling of XCC complexes using BILBO‐MD reveals the coexistence of compact and elongated conformations within the ensemble. The relative population of each conformational state is indicated, together with the corresponding radii of gyration (R g ) and maximum dimensions (D max). FIGURE S7. χ2 values for MES fit in dependence to the number of selected conformers for the [file PRO-35-e70649-s001.pdf]

Figure S1. All atom and *ab initio* modeling of *At*Xyn11A. (A) *Ab initio* reconstructions of *At*Xyn11A generated using GASBOR and the corresponding NSD. (B) Five independent models of *At*Xyn11A superimposed into the catalytical domain.

Figure S2. All-atom modeling of the CC using DADIMODO. Five independent models of the CC_1 (A) or CC_2 (B) assemblies generated with DADIMODO, shown as Cα ribbon representations superimposed on the JoIn core along with the corresponding χ^2 values are reported in the left panel. The Cα NSD matrix is shown in the right panel. C) Distances of 5 models displayed with mean and SD as displayed in figure 4.

Figure S3. *Ab initio* reconstructions generated using GASBOR. Panels show five independent GASBOR models and the most representative envelope for CC_1 (A) and CC_2 (B), together with the corresponding NSD values

Figure S4. In silico shape simulations of CC_1 and CC_2 generated using an in-house MATLAB workflow. (A,B) Representative AFM-like top-view projections of CC_1 (A) and CC_2 (B) for a subset of randomly sampled surface-bound orientations. Each panel shows the theoretical projected particle footprint after rasterization and morphological processing to approximate AFM tip broadening, assuming a nominal tip radius of 1 nm. The images illustrate the variability in projected surface coverage arising from different adsorption geometries. (C) Histograms of solidity (defined as the ratio of particle area to convex hull area) obtained from 2000 randomly sampled orientations for CC_1 and CC_2. CC_1 exhibits slightly lower solidity values, consistent with a more elongated projected shape, whereas CC_2 shows higher solidity values, indicating a more compact footprint.

Figure S5. All-atom modeling of the XCC complexes using DADIMODO. Five independent models were generated and superimposed within the JoIn scaffold, with the most representative model shown in bold. For each complex, the residual corresponding to the most representative model is displayed.

Figure S6. Modeling of XCC complexes using BILBO-MD. Compact and elongated conformations coexist within the ensemble. The relative population of each conformational state is indicated, together with the corresponding radii of gyration (Rg) and maximum dimensions (Dmax).

Figure S7. **X^2 values for MES fit in dependence to the number of selected conformers.** 6 XCC assemblies show a net decrease of the X^2 after 2 conformers followed by a plateau when more conformers are integrated.

Figure S8. SDS-Page of the purified complexes and the free enzymes.

Figure S9. Guinier analysis of all assemblies and free enzymes used in this study. The inset displays the corresponding residuals of the fit, indicating the quality of the Guinier approximation within the selected q-range.

Figure S1

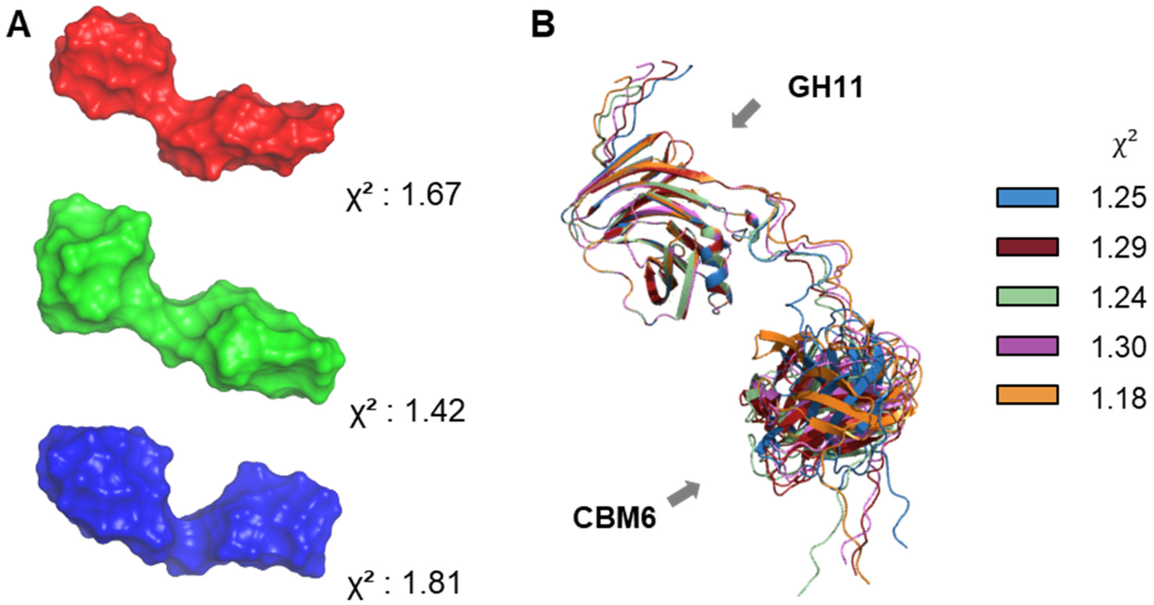

Figure S2

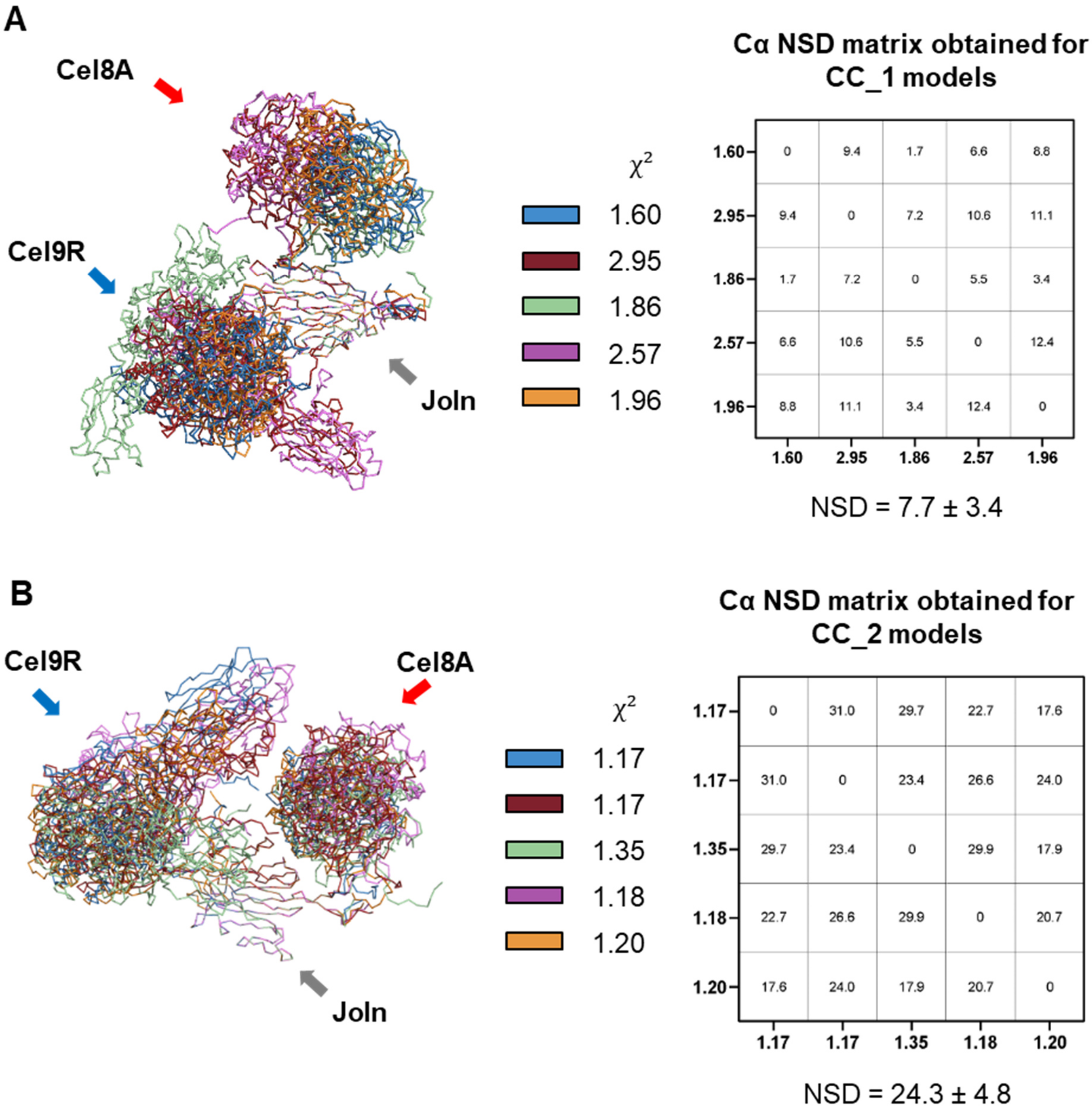

C

Distance Cel8A-Cel9R	Model 1	Model 2	Model 3	Model 4	Model 5	Mean ± SD
**CC_1** (D251-E1023)	88	82	86	88	84	86 ± 3
**CC_2** (D332-E1023)	74	66	64	72	65	68 ± 4

Figure S3

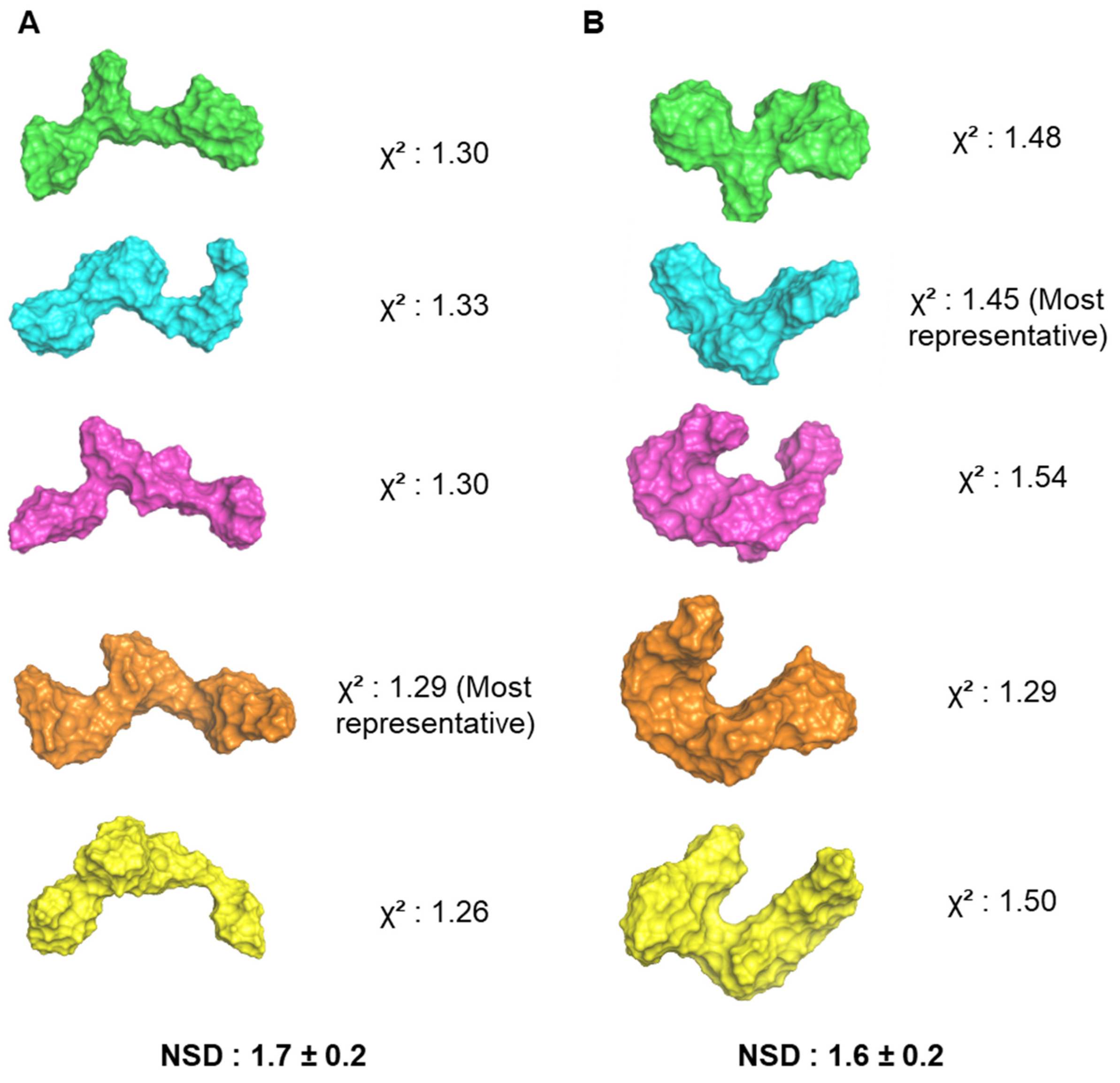
A
B
χ² : 1.30
χ² : 1.33
χ² : 1.30
χ² : 1.29 (Most representative)
χ² : 1.26
NSD : 1.7 ± 0.2
χ² : 1.48
χ² : 1.45 (Most representative)
χ² : 1.54
χ² : 1.29
χ² : 1.50
NSD : 1.6 ± 0.2

Figure S4

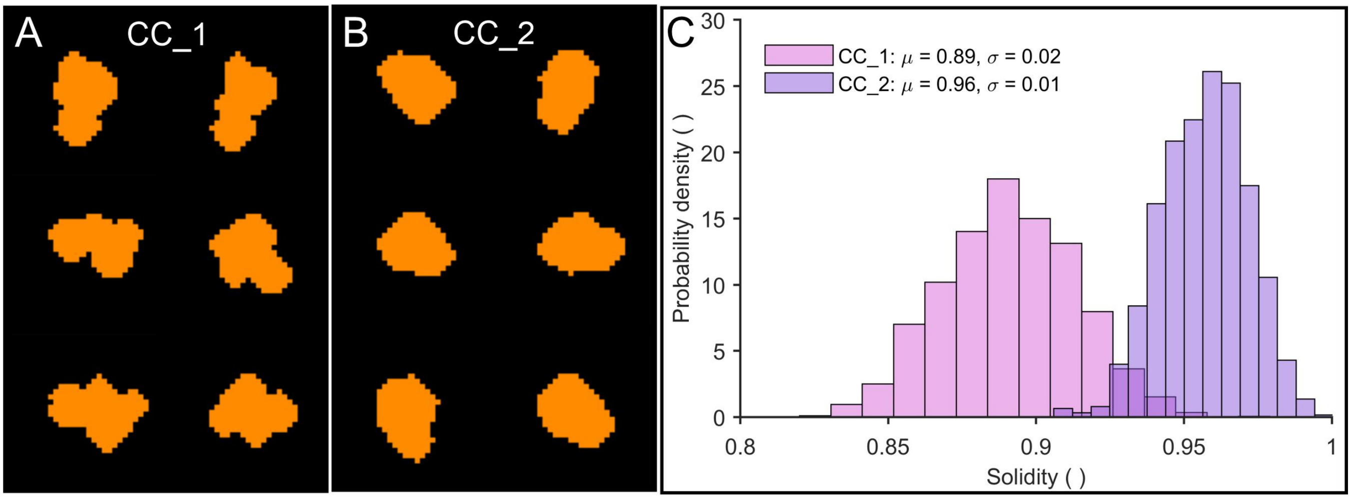

A
CC_1
B
CC_2
C
CC_1: μ = 0.89, σ = 0.02
CC_2: μ = 0.96, σ = 0.01
Probability density ( )
Solidity ( )
0
5
10
15
20
25
30
0.8
0.85
0.9
0.95
1

Figure S5

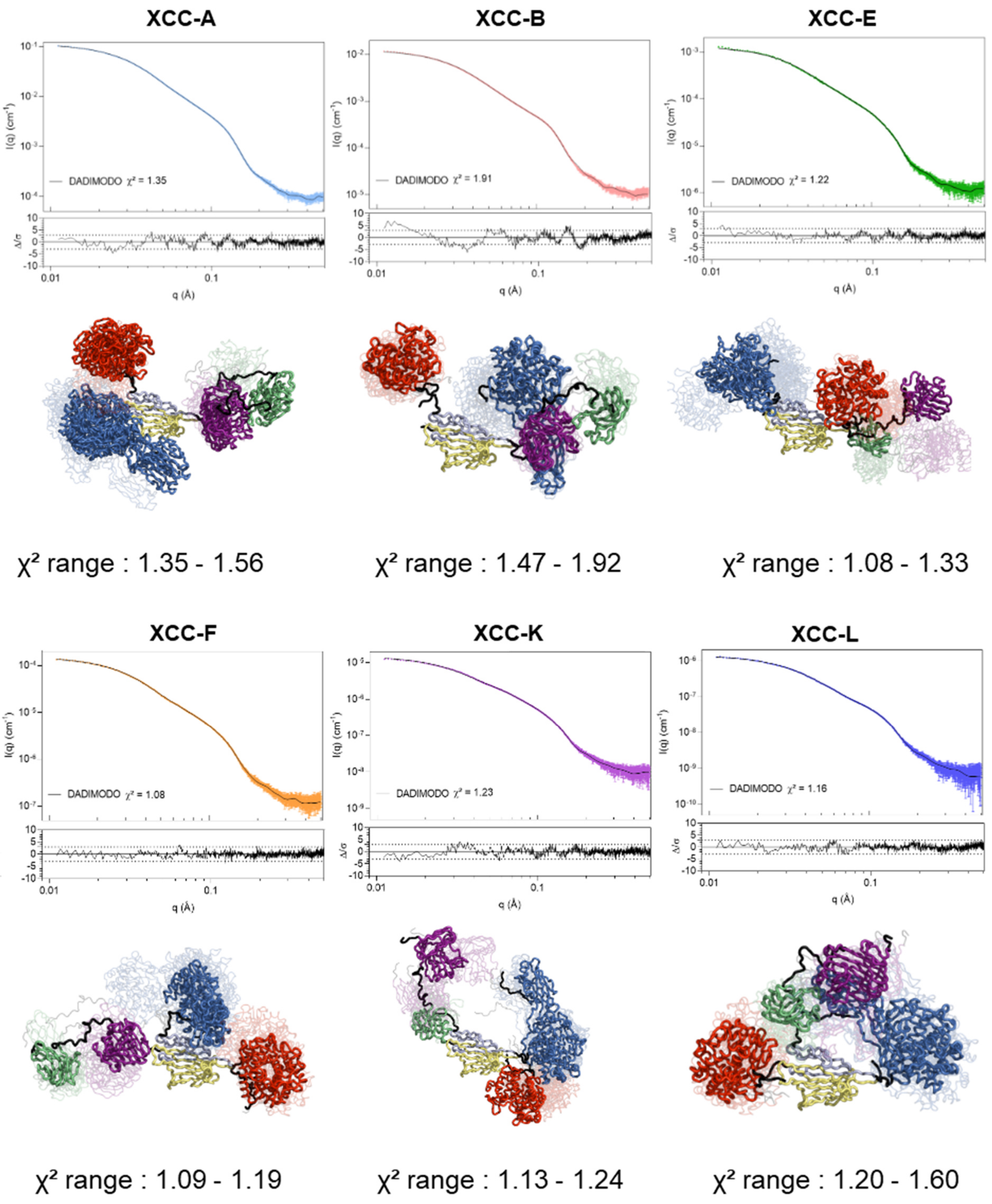
XCC-A
DADIMODO χ² = 1.35
χ² range : 1.35 - 1.56
XCC-B
DADIMODO χ² = 1.91
χ² range : 1.47 - 1.92
XCC-E
DADIMODO χ² = 1.22
χ² range : 1.08 - 1.33
XCC-F
DADIMODO χ² = 1.08
χ² range : 1.09 - 1.19
XCC-K
DADIMODO χ² = 1.23
χ² range : 1.13 - 1.24
XCC-L
DADIMODO χ² = 1.16
χ² range : 1.20 - 1.60
I(q) (cm⁻¹)
Δ/σ
q (Å)

Figure S6

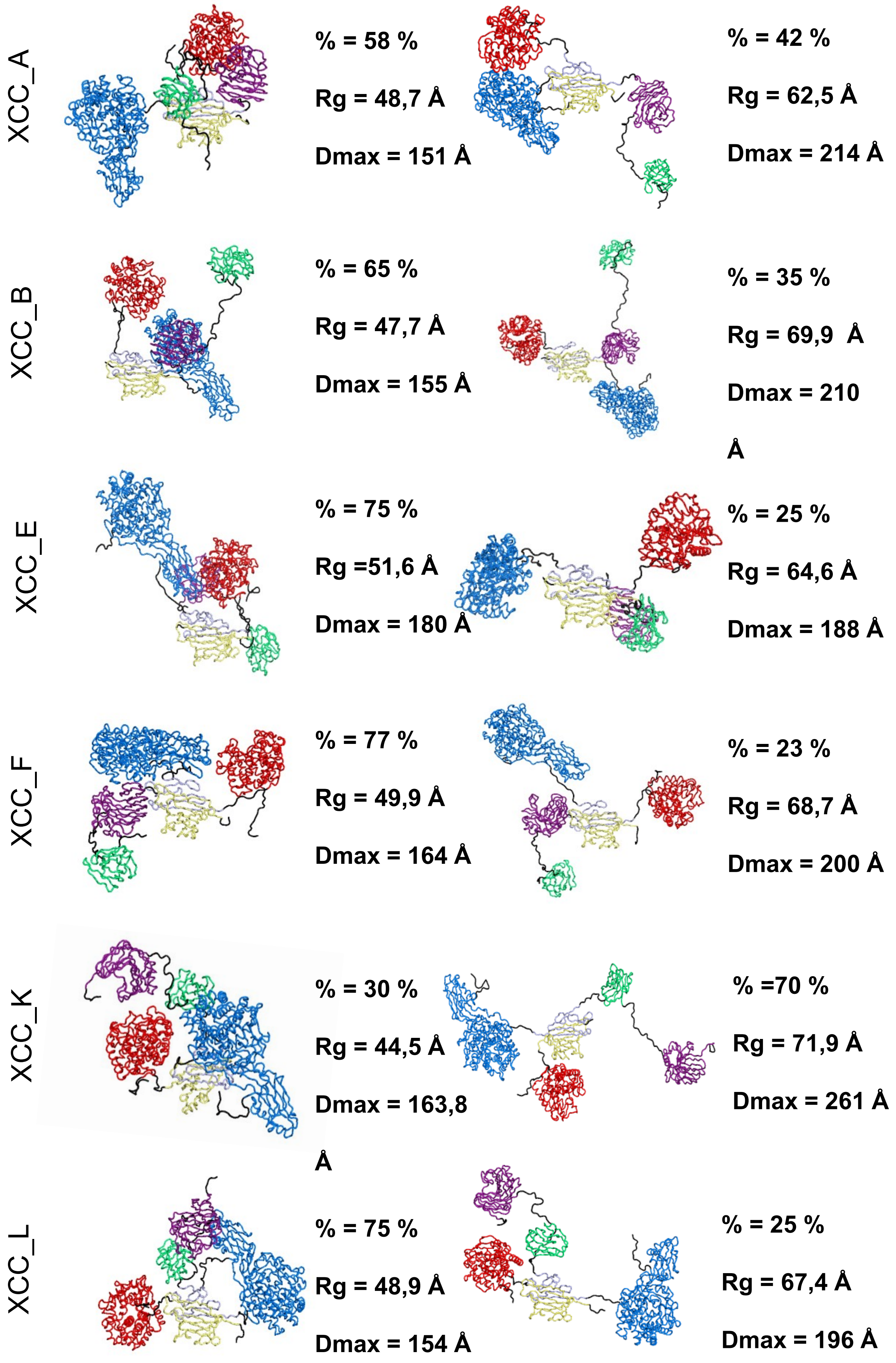
XCC_A
% = 58 %
Rg = 48,7 Å
Dmax = 151 Å
% = 42 %
Rg = 62,5 Å
Dmax = 214 Å
XCC_B
% = 65 %
Rg = 47,7 Å
Dmax = 155 Å
% = 35 %
Rg = 69,9 Å
Dmax = 210 Å
XCC_E
% = 75 %
Rg =51,6 Å
Dmax = 180 Å
% = 25 %
Rg = 64,6 Å
Dmax = 188 Å
XCC_F
% = 77 %
Rg = 49,9 Å
Dmax = 164 Å
% = 23 %
Rg = 68,7 Å
Dmax = 200 Å
XCC_K
% = 30 %
Rg = 44,5 Å
Dmax = 163,8 Å
% =70 %
Rg = 71,9 Å
Dmax = 261 Å
XCC_L
% = 75 %
Rg = 48,9 Å
Dmax = 154 Å
% = 25 %
Rg = 67,4 Å
Dmax = 196 Å

Figure S7

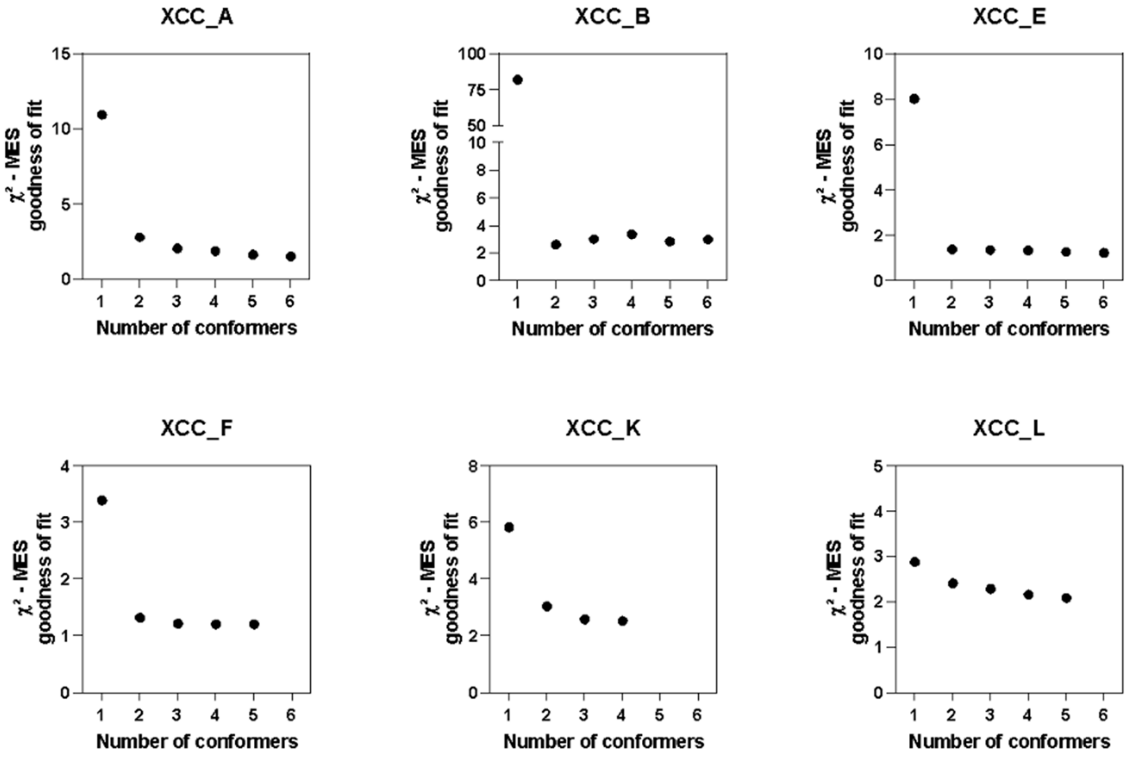
XCC_A
XCC_B
XCC_E
XCC_F
XCC_K
XCC_L
χ² - MES goodness of fit
Number of conformers

Figure S8

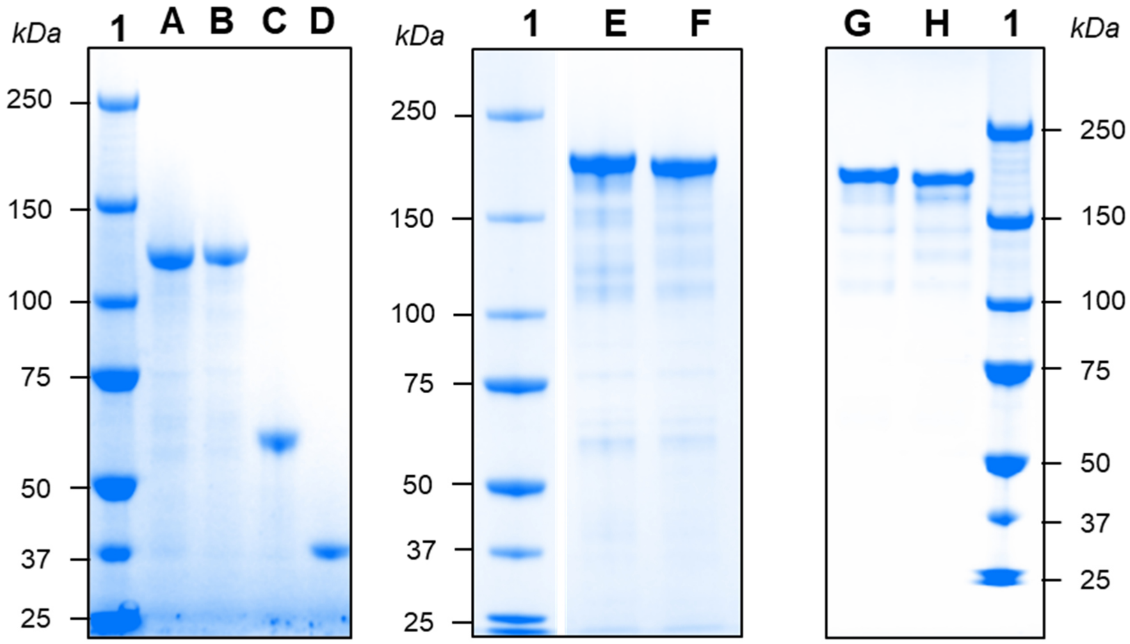

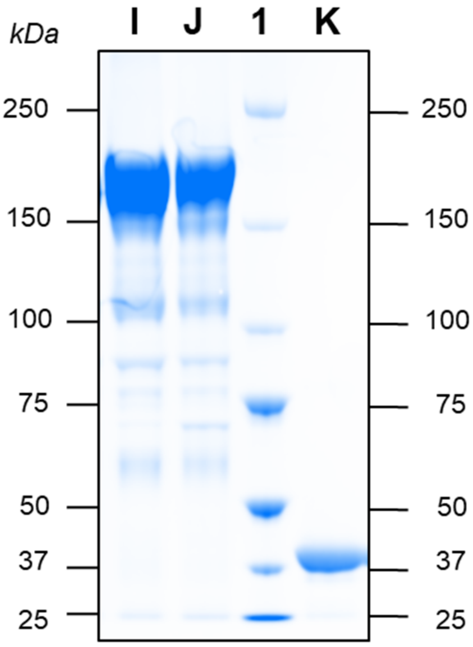

Legend

- 1 : Ladder -Precision plus protein ladder Biorad
- A: CC_1 (2,5 µg)
- B: CC_2 (2,5 µg)
- C: Cel9R (2,5 µg)
- D: Cel8A (2,5 µg)
- E: XCC-A (2,5 µg)
- F: XCC-B (2,5 µg)
- G: XCC-E (2,5 µg)
- H: XCC-F (2,5 µg)
- I: XCC-K (> 10 µg)
- J: XCC-L (> 10 µg)
- K: Xyn11A (>10 µg)

Figure S9

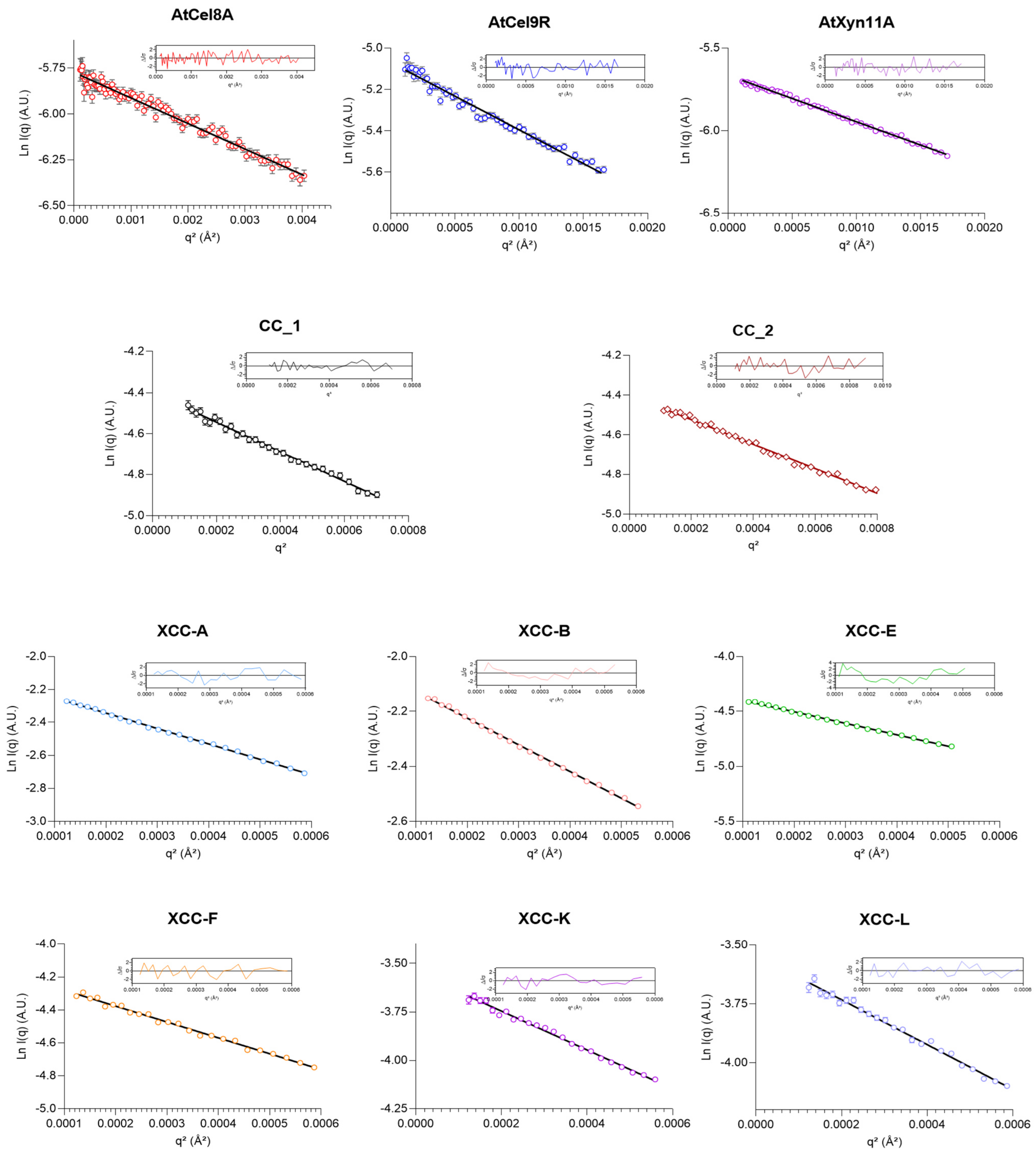
AtCel8A
AtCel9R
AtXyn11A
CC_1
CC_2
XCC-A
XCC-B
XCC-E
XCC-F
XCC-K
XCC-L
Ln I(q) (A.U.)
q² (Å²)
q²
Δ/σ
